# Monitoring radiation exposure through skin swab multi-omic profiling

**DOI:** 10.1371/journal.pone.0354734

**Published:** 2026-08-05

**Authors:** Geraldine Vitry, Jerry Angdisen, Pauline Arriaga, Shawn Irgen-Gioro, Megan A. Sawant, Daniel C. Vuong, Peter Ilhardt, Jacques Fehr, Bartosz Cwikla, Brian Ponnaiya, Jamie L. Inman, Jian-Hua Mao, Antoine M. Snijders, Shazia Hamid, David Caballero-Lima, Guy Garty, Karyn Apfeldorf, Evagelia C. Laiakis

**Affiliations:** 1 Department of Oncology, Lombardi Comprehensive Cancer Center, Georgetown University Medical Center, Washington, District of Columbia, United States of America; 2 Data Analytics, Areté Associates, Northridge, California, United States of America; 3 Currently at: Harvard University, Cambridge, Massachusetts, United States of America; 4 Currently at: Amentum, NASA Johnson Space Center, Houston, Texas, United States of America; 5 Radiological Research Accelerator Facility, Columbia University, Irvington, New York, United States of America; 6 Labskin LTD, York, United Kingdom; 7 Center for Radiological Research, Columbia University Irving Medical Center, New York, New York, United States of America; 8 Lawrence Berkeley National Laboratory, Berkeley, California, United States of America; 9 Currently at: Biosciences and Biotechnology Division, Lawrence Livermore National Laboratory, Livermore, California, United States of America; 10 ATCC, Manassas, Virginia, United States of America; 11 Microsapient, Freelance, York, United Kingdom; 12 Department of Radiation Medicine, Georgetown University Medical Center, Washington, District of Columbia, United States of America; 13 Department of Biochemistry and Molecular & Cellular Biology, Georgetown University Medical Center, Washington, District of Columbia, United States of America; 14 Center for Metabolomic Studies, Georgetown University, Washington, District of Columbia, United States of America; Xiangtan University, CHINA

## Abstract

Exposure to ionizing radiation poses major health risks across medical, occupational, and spaceflight settings, driving the need for rapid, non-invasive biodosimetry tools. As the body’s most accessible organ and the most frequent site of radiation injury, the skin represents a promising interface for monitoring exposure. Using colonized human skin equivalents (coHSE; 0 Gy n = 8, 1 Gy n = 6, 4 Gy n = 6) and mice (n = 6/group) models, we performed multi-omic profiling, integrating metabolomics, lipidomics, and metagenomics, on skin swab samples collected after exposure to 0, 1, or 4 Gy of x-rays. We identified two distinct metabolite panels: one discriminating irradiated from non-irradiated skin, and another distinguishing dose-specific response. These panels included conserved radiation-responsive metabolites (e.g., uric acid, xanthine, taurine) and skin-specific markers associated with barrier integrity (e.g., proline, arginine). Diacylglycerol network enrichment and shifts in radioprotective microbial taxa, including *Lachnospiraceae* and *Lactobacillales,* further supported a repair-driven molecular response. These data support the feasibility of skin swab signatures for non-invasive exposure classification, providing a molecular and microbial framework for skin based monitoring measure development and motivating validation in human cohorts for real-world biodosimetry.

## Introduction

Since the discovery of radioactivity in the late 19th century, the use of radiation across various domains, such as nuclear energy, medical diagnostics, and cancer therapy, has led to a steady increase in human exposure to ionizing radiation (IR). Accidental releases, reactor failures, and radiological incidents, whether unintentional or deliberate, have underscored the need for rapid radiation dose assessment. Events such as the Chernobyl and Fukushima-Daiichi nuclear accidents, or localized contamination episodes like Goiânia in Brazil, illustrate how technical, natural, or human factors can cause lasting radiological, biological, and environmental consequences [1,2]. In parallel, concerns about the potential use of radiological dispersal or improvised nuclear devices in populated areas highlight the urgency of developing scalable biodosimetry strategies for diverse exposure scenarios. Meanwhile, expanding aeronautics and space activities expose individuals to cosmic radiation, with nuclear-powered propulsion systems, key enabling technologies to future planetary exploration, representing an additional source of radiation in the coming decades [3–6].

IR elicits a wide spectrum of biological effects, from transient cellular stress to irreversible tissue damage, organ failure, and death, depending on dose, dose rate, and radiation quality. Accordingly, the development of rapid and reliable biodosimetry capable of estimating absorbed dose through biological markers has become critical to radiological emergency response, occupational safety, and personalized medicine. However, current biodosimetry approaches face critical limitations in scalability, accessibility, and time to result.

According to the Biomedical Advanced Research and Development Authority (BARDA) recommendations, biodosimetry results should ideally be available in less than 15 min and cover a dose range of 0.5–10 Gy [4]. The gold standard approaches include cytogenetic techniques such as dicentric chromosome analysis and the cytokinesis-block micronucleus assay (CBMN). Theses assays, while highly accurate, involve labor-intensive, time-consuming techniques that typically require specialized laboratory infrastructure and several days to generate results, limiting their utility for rapid triage and field deployment during large-scale exposure events. Furthermore, these approaches also provide limited phenotypic insight that could guide preventive or therapeutic countermeasures. Similarly, alternative blood-based molecular and protein biomarkers methods, such as lymphocyte depletion kinetics via complete blood count (CBC), premature chromosome condensation (PCC), γ-H2AX foci formation, and physical dosimetry using Electron Paramagnetic Resonance (EPR) and Optically Stimulated Luminescence (OSL) of the teeth, require invasive sampling and may not be readily deployable for large-scale or longitudinal monitoring [6–17]. Therefore, there is an unmet need to develop alternative non-invasive biodosimetry approaches rapidly deployable, scalable, and capable of reflecting not only radiation dose but also biological response trajectories

Based on mass spectrometry, metabolomic analyses enable the rapid identification and the measurement of all small molecules of <1 kDa, that reflect the physiological phenotype associated with a given sample. Over the past two decades, radiation metabolomics and lipidomics have extensively profiled alterations in accessible biofluids, including serum, urine, and saliva, identifying reproducible panels of radio-responsive metabolites conserved across species and sample types, supporting their deployment in diverse scenarios [18–21]. When combined with other –omics results, metabolomics can provide an overview not only of the effects of dose and tissue injury, but also of novel pathways for therapeutic intervention. Rapid advancements in digital technologies and wearable biosensors are expanding the capabilities of diagnosis tools to longitudinal, scalable, real-time health monitoring [22]. The skin, being the body’s largest and most accessible organ, may offer an innovative solution in this context.

The skin is highly responsive to radiation. Over 90–95% of radiotherapy patients develop acute radiation dermatitis, and 10–30% experience chronic effects such as fibrosis, atrophy, and telangiectasias [23,24]. In accidental or intentional exposures (>2 Gy), cutaneous radiation syndrome (CRS) occurs in 20–50% of individuals [25], with symptoms ranging from erythema and blistering to necrosis [26–29]. Importantly, the severity of cutaneous injury is formally recognized as a prognostic indicator by the IAEA, WHO, and U.S. REMM [30–32] correlating with total absorbed dose and systemic outcomes such as hematopoietic failure and multi-organ dysfunction, is incorporated into the Medical Treatment Protocols for Radiation Accident (METREPOL) consensus for clinical grading of irradiated victims and outcome prediction [33]. In addition, the skin microbiota are highly sensitive to environmental conditions, and alterations in the skin microbiome have been shown to reflect various clinical outcomes including wound healing and recovery from radiation-induced dermatitis [34–40]. We recently demonstrated that skin swabs collected from bioengineered human skin equivalents colonized with natural microbial communities and the mouse skin displayed similar taxonomic and metabolomic features in response to radiation exposure [41]. Operationally simple, skin swabs offer a minimally invasive, low training-burden sampling strategy compatible with diverse analytic platforms that can be repeated frequently, enabling longitudinal assessment. Unlike many biofluid-based approaches, swabs capture responses at a highly accessible and most directly exposed tissue interface and simultaneously measure host metabolites/lipids and microbiome shifts, enabling multi-layered signatures for exposure assessment.

In the effort of developing simple, non-invasive skin-based biodosimetry method, the objective of this study was to evaluate the skin as a novel, accessible, and biologically informative tissue for radiation biodosimetry by characterizing dose-dependent molecular and microbial signatures following IR in skin swab samples. We hypothesized that the skin harbors conserved and functionally relevant alterations in metabolites, lipids, and microbial taxa that can reflect radiation dose and potentially reveal insights into systemic health outcomes. To test this hypothesis, we conducted a multi-omics analysis integrating metabolomics, lipidomics, and 16S rRNA-based microbiome profiling using skin swab samples collected from mouse models exposed to 0, 1, or 4 Gy of total body irradiation. These radiation doses were selected to reflect exposure levels relevant to radiation emergency scenarios, spanning a biologically and clinically relevant range recognized in biodosimetry frameworks. This dose interval enables evaluation of dose-dependent molecular responses and assessment of the capacity of skin-based multi-omic profiling to detect and discriminate radiation exposure under realistic and translationally relevant photon exposure conditions. To assess translational relevance and conservation of response, complementary analyses were also performed on full-thickness bioengineered human skin equivalents colonized with natural skin microbial communities from human donors (coHSE) and subjected to the same radiation doses. Multivariate statistics, pathway enrichment, and correlation analyses using the R mixOmics DIABLO framework, a supervised multiblock integration method using sparsity constraints (sparse partial least square differential analysis, sPLS-DA) for identifying correlated and discriminative features across omics datasets, were employed to identify radiation-responsive features, and overlap with previously reported radioresponsive molecular and microbial biomarkers. In particular, DIABLO enables integration of multi-omic datasets and identification of correlated biomarker panels. To balance exploratory multi-omic feature discovery with assessment of generalizability, we implemented a two-stage analytical design in which DIABLO was used to identify candidate radiation-responsive features in a discovery cohort, followed by independent validation of these signatures in a separate mouse cohort not used for feature selection or model fitting. Given the importance of natural moisturizing factor (NMF) on skin hydration and barrier function, we assessed the dose responsiveness of its key components including lactic acid, urea, pyrrolidone carboxylic acid (PCA), urocanic acid (UCA), and byproducts of filaggrin degradation (e.g., serine, glycine, alanine, histidine, arginine, glutamic acid, aspartic acid, threonine, proline).

## Materials and methods

### Human skin equivalents

Commercially available full-thickness human skin equivalents (HSE) were obtained from Labskin (Deepverge, UK). The production method of this model can be found in Holland et al., 2008 [42]. HSEs were shipped at day 12 of air-liquid interface maturation (day ALI 12) in semi-solid media and equilibrated overnight at 37°C and 5% CO_2_ in 12-well plates containing 1 mL of maintenance medium upon reception. The day after arrival, HSE were inoculated with natural skin microbiota isolated from healthy adult volunteer recruited by American Type Culture Collection (ATCC) under IRB-approved protocol (Pro00067707). Skin microbial communities were collected from both cheeks of 38 donors using a scrub-wash method (ATCC protocol) [43], and pooled to increase colony-forming unit (CFU) yields. HSEs were colonized using human skin microbiome samples collected from human donors, as previously described [44], with varied age, sex, and ethnicity ensuring representative diversity. To ensure that colonized HSE (coHSE) reflected representative human skin microbial diversity while maintaining donor-level biological variability, colonization was performed using microbiome samples derived from 11 independent human donors resulting in 20 total inoculations distributed across experimental radiation dose groups (0 Gy: n = 8; 1 Gy: n = 6; 4 Gy: n = 6). Each experimental condition included microbiomes from multiple donors, and several donors contributed samples across different dose groups, reducing potential donor-specific bias (Supplemental S1 Table). The donor cohort included both female (63.6%) and male (36.4%) participants and spanned two adult age ranges (20–40 years: 36.4%; 40–60 years: 63.6%) (Supplemental S1 Table). Although females were more represented overall and male donors were primarily represented in the younger age group, inclusion of multiple independent donors per condition ensured that results were not driven by a single donor or demographic subgroup.

Pooled microbial suspensions were washed with sterile deionized water and centrifuged twice to remove detergents and debris. Each HSE was inoculated with 10 µL of microbial suspension (at 1x10^6^ CFU/mL which translates to 1x10^4^ CFU/cm^2^) one day before irradiation (at day 14 of HSE maturation). A slightly larger sham group was included in the coHSE model to improve characterization of baseline variability inherent to human-derived microbial communities and to provide a robust reference for downstream comparative and integrative analyses. The next day, coHSEs were exposed to x-rays at 0 Gy (n = 8), 1 Gy (n = 6), or 4 Gy (n = 6), 24h after inoculation, in a XRAD 320 irradiator (320 kVp, 12.5 mA) fitted with a beam-conditioning filter (0.75 mm Tin + 0.25 mm Copper + 1.5 mm Aluminum) providing a specific beam Half Value Layer (HVL) of ≅ 4 mm Cu at 50 cm from the source. The dose rate was 0.85 Gy/min and dosimetry was performed using a NIST-traceable ionization chamber (Radcal 10x6-6, Monrovia CA) calibrated to air kerma. This irradiation system operates within established X-ray range for in vitro radiation biology and skin irradiation studies. Delivered dose was verified using NIST-traceable dosimetry, ensuring accurate and reproducible absorbed dose (Gy), the biologically relevant parameter reflecting energy deposition in tissue.

Swab sample collection was standardized and consisted of 10 gentle rounds over the surface of each HSE construct for a time duration of 10 seconds. Swab samples were collected at 1, 3, and 7 days post-irradiation, snap frozen in liquid nitrogen and stored at −80˚C in 1.5 mL tubes until further processing.

### Animal procedures

All animal procedures were approved by the Lawrence Berkeley National Laboratory IACUC (Protocol #270036, OLAW Assurance D16-0031/A3054-01). Male and female C57BL/6J mice (9–11 weeks old) were obtained from Jackson Laboratories and acclimated for two weeks before radiation exposure. Serving as a discovery cohort (see “ Statistical analysis” description section below): mice were randomly assigned to dose groups with equal sex repartition: 0 Gy (sham, n = 6), 1 Gy (n = 6), and 4 Gy (n = 6), and underwent total body irradiation, placed on a rotating platform (4 RPM) at 50 cm from the source, in a XRAD 320 irradiator (300 keV/10mA) fitted with a 0.5 mm Cu filter (HVL ≅ 1.7 mm Cu), at a dose rate of 1.3 Gy/min. Dosimetry was performed using a RadCal ionization chamber (Radcal 10X6-0.18) and gafchromic film. This system also operates within appropriate X-ray voltage ranges for small animal irradiation and skin radiation studies. Differences in filtration and dose rate reflect facility-specific equipment configurations. However, in both experimental systems, delivered dose was verified using NIST-traceable dosimetry, ensuring accurate and reproducible absorbed dose (Gy), thereby enabling valid comparison of dose-dependent biological responses across models. Skin swabs were collected from a 1.5 × 6 cm area on the dorsal skin, which was shaved at least 24 hours prior to irradiation to allow initial microbial recovery, with a minimum of 48 hours between shaving and the first post-irradiation sample collection at 1, 3, and 7 days. The timing of shaving was prospectively optimized during experimental planning and incorporated into a standardized operational procedure to minimize transient perturbations of the skin barrier and microbiome while ensuring consistent and reproducible sampling conditions across all animals. Mice were housed in standardized conditions on Sani-Chips bedding (P.J. Murphy). Swab samples were snap frozen in liquid nitrogen and stored at −80˚C in 1.5 mL tubes until furth1er processing.

### Untargeted metabolomic profiling

Metabolites were extracted as previously described [45]. Briefly, each swab was incubated in 50% LC-MS grade ethanol with internal standards (30 µM 4-nitrobenzoic acid, Sigma cat# 72910; 2 µM debrisoquine sulfate Sigma cat# D1306; 5 µM chlorpropamide Sigma cat# C1290 final concentrations) for 2 hours at 4 °C. Extracts were transferred to clean Eppendorf tubes, dried in a speed vac at room temperature, and resuspended in 50 µL of 0.1% formic acid in water. Samples were filtered (Bio-Inert® Membrane 0.2 µm, aqua Cat# ODM02C34) and transferred to 250 µl vials (Thermo fisher #C4011-13) for subsequent liquid chromatography (LC) time-of-flight MS analysis. A pooled quality control (QC) sample was prepared by combining 5 µL from each sample. Samples were run an LC-MS using a Waters Acquity Ultra Performance Liquid Chromatography (UPLC) with a BEH C18 1.7 µm, 2.1 × 100 mm column coupled to a Xevo® G2-S quadrupole time-of-ﬂight (QTOF) MS (Waters, Milford, MA, USA) with data independent acquisition (DIA). Positive and negative electrospray ionization (ESI+ and ESI–) was used with leucine enkephalin ([M + H]+ = 556.2771, [M-H]− = 554.2615) as lock-mass. Operating conditions for ESI were: capillary voltage 2.75 kV, cone voltage 30 V, desolvation temperature 500 ◦C, desolvation gas ﬂow 1000 L/Hr. Mobile phases were water with 0.1% formic acid (A) and acetonitrile with 0.1% formic acid (B). The gradient was set to 5% B for 1.12 min, followed by an increasing proportion of B to 99.5% at minute 6.4 and a plateau for the remaining 3.6 min. Column temperature was kept at 40 °C, and flow rate was set to 0.4 mL/min. Blanks and QC samples were run every 10 samples. Candidate metabolites were validated to a metabolomics standards initiative (MSI) level 1 where we matched the accurate *m/z*, retention time, and tandem MS (5–50 V ramping collision energy) fragmentation patterns against pure standards, or level 2, where fragmentation patterns were matched using online library (e.g., HMDB, LIPID MAPS, METLIN) accessible through the Progenesis QI software (see S2 Table) [46,47].

### Targeted lipidomic profiling

Lipids were extracted with a biphasic method as previously described [48]. Each swab sample was incubated in 70% LC-MS grade ethanol containing lipid standards (1 µg/ml EquiSPLASH® LIPIDOMIX, Avanti cat# 330731–1EA; 2.5 µg/ml 18:1 Chol (D7) ester Avanti cat# 700185M-1MG; 5 µg/ml 15:0–18:1-d7-PA Avanti cat# 791642C-1MG) for 2 hours at 4˚C. Samples were transferred to a new clean 1.5 ml siliconized tube (CPLab Safety, cat# BP-4167SLS50) and one ml of chloroform/methanol/water (1:2:0.8) was added to each sample for biphasic extraction. Samples were centrifuged for 10 min at maximum speed at 4°C, and the lower phase was placed in a new fresh 1.5 ml siliconized tube. Samples were dried in a speed vac at room temperature and resuspended in 200 µl of methanol/isopropanol/acetonitrile (9.65v/1v/3v). Sample were filtered and transferred to 250 µl glass vials (Thermo fisher #C4011-13) for MS analysis. A pooled QC sample was prepared by combining 5 µL from each sample. Lipids (5 µL) were separated on a Xbridge amide column (3.5 µm, 4.6 × 100 mm, 35 °C) (Waters, Milford, MA, USA) and analyzed in a Sciex QTRAP 5500 Mass Spectrometer (Sciex, Framingham, MA, USA), using mobile phases C (95% acetonitrile/5% water + 10 mM ammonium acetate) and D (50% acetonitrile/50% water + 10 mM ammonium acetate). Lipids were detected by multiple reaction monitoring (MRM) transitions in both positive and negative ionization modes (listed in Supplementary S1 File). Gradient elution transitioned as follow: initial gradient 100% C, 3.0 min 99.9% C, 3.0 min 94% C, 4.0 min 25% C, 6.0 min 0% C, 6.0 min equilibrate back to 100% C, at a ﬂow rate of 0.7 mL/min. Source and gas settings were as follows: temperature = 550 ◦C, nebulizing gas = 50 and heater gas = 60, curtain gas = 30, CAD gas = medium, ion spray voltage = 5.5 kV in positive mode and −4.5 kV in negative mode. Blanks and QC samples were run after every 10 samples. Lipid classes analyzed included: cholesteryl esters (CE), cholesterol, ceramides (Cer), hexosylceramides (HexCer), lactosylceramides (LCER), dihydroceramides (DCER), sphingomyelins (SM), acylcarnitines, diacylglycerides (DAG), triacylglycerides (TAG), monoacylglycerides (MAG), free fatty acids (FFA), phosphatidic acids (PA), lysophosphatidic acids (LPA), phosphatidylcholines (PC), ether-linked phosphatidylcholines (ePC), lysophosphatidylcholines (LPC), phosphatidylinositols (PI), lysophosphatidylinositols (LPI), phosphatidylethanolamines (PE), ether-linked phosphatidylethanolamines (ePE), lysophosphatidylethanolamines (LPE), phosphatidylglycerols (PG), and phosphatidylserine (PS) [49]. Lipid species were annotated using standard lipid class abbreviations (e.g., TAG, CL). Instrument-specific identifiers and charge annotations (e.g., “(-2)”) were retained in figures for consistency with lipidomics software outputs and are defined in figure legends.

### Microbiome profiling (16S sequencing)

Swab samples collected from mice or coHSEs were preserved in DNA/RNA Shield (Zymo Research, Fisher scientific #50-125-1706) at −80°C. Frozen swab samples were shipped on dry ice to Novogene for 16S sequencing.

### Data processing

Untargeted metabolomic data were processed with the Progenesis QI software (Nonlinear Dynamics, Newcastle, U.K.) for peak alignment and peak picking. Adducts for compound deconvolution were set to M + H, M + H–H2O, M + NH4, M + Na (ESI+) or M-H, M-H2O–H (ESI−). Peak alignment was performed using a software chosen QC chromatogram and normalization references applying a log-ratio scale factor calculated from all detected compounds in each sample (“normalize to all compounds” function). Putative identifications for spectral features were assigned using monoisotopic mass (±8 ppm error), and theoretical fragmentation patterns from the human metabolome database (HMDB), LIPID MAPS, and the METLIN MS/MS library [46,47].

For targeted lipidomics, raw data and peak areas were visually inspected using the MultiQuant v.2.0 software (Sciex, Framingham, MA, USA) and data were then exported to Microsoft Excel. Lipids present in the QC sample with a coefficient of variation >25% were removed.

Microbiome (16S rRNA) data were processed and analyzed using QIIME2 for taxonomic profiling. Amplicon sequence variants (ASVs) were inferred with the Divisive Amplicon Denoising Algorithm 2 (DADA2) pipeline, and taxonomy was assigned using QIIME’s pretrained naïve Bayes classifier trained on the GreenGenes 16S reference database. Taxa with near-zero variance were removed, and near-constant variables were further filtered by an interquartile range variance filter of 40%. Differential abundance analysis was performed using DESeq2 (R package version 6.1.1) [50].

### Multi-omic Integration

Metabolomic, lipidomic, and metagenomic datasets collected from the same mouse or coHSE were integrated using the mixOmics methods and R packages [version 4.3.2 (2023-10-31)] [51]. DIABLO (Data Integration Analysis for Biomarker discovery using Latent variable approaches for Omics studies), a supervised multiblock sPLS‑DA-based integration method was used to identify correlations between datasets measured across same samples. DIABLO constructs latent components that maximize covariance across multiple omic blocks while enforcing discriminative power for predefined categorical outcomes (radiation doses). The detailed pipeline can be found at https://mixomics.org/mixdiablo/diablo-tcga-case-study/. Briefly, a design matrix was used to specify pairwise correlations between datasets, controlling the trade-off between integration and prediction accuracy. Following mixOmics recommendations, a low design value (0.1) was selected to prioritize discrimination of radiation dose groups over maximizing feature correlations across datasets, as higher design values (≥ 0.5) tend to enhance correlation but reduce the predictive ability of the model. During model fitting, a LASSO (least absolute shrinkage and selection operator) penalization was applied to the loading vectors via sparse partial least squares discriminant analysis (sPLS-DA) to select features consistent with both the design constraints and discriminative power [52,53]. The top discriminant features, those contributing most strongly to the latent components and maximizing pairwise correlations, were ranked by loading strength and visualized using sample projection plots, correlation circle plots, circos networks, variable loading plots, and ROC curves to highlight multi-omic signatures distinguishing irradiated from non-irradiated samples and dose-dependent responses.

Over representation analyses (ORA) were conducted in MetaboAnalyst 6.0 (Enrichment Analysis module) using the Small Molecule Pathway Database (SMPDB), the Kyoto Encyclopedia of Genes and Genomes (KEGG) databases, and the “1,250 sub chemical class metabolite sets or lipid sets” library to analyze enriched pathways and lipid classes in metabolomic and lipidomic datasets [54]. MetaboAnalyst 6.0 (biomarker analysis module) was also used to analyze and generate ROC curves. The Lipidmap BioPan online software was used to analyze lipid reaction network enrichment [55].

### Statistical analysis

#### General.

Because radiation responses exhibit temporal dynamics, samples collected at days 1, 3, and 7 post-irradiation were pooled for primary dose discrimination analyses to identify molecular and microbial signatures that are conserved across early post-exposure time points and primarily reflect radiation dose rather than time-dependent biological recovery processes. This time window reflects operational biodosimetry scenarios where rapid exposure classification is required regardless of the exact time since exposure within the early response phase. By reducing the influence of temporal variability, this strategy enables identification of biodosimetric features capable of discriminating exposure level independently of post-exposure temporal progression during the first week following irradiation.

#### Data normalization, drift correction, and batch correction.

Metabolite intensity tables (normalized to total compounds) exported from Progenesis QI were processed in Python (v3.9) using custom scripts. Data processing included global normalization, quality control–based signal drift correction, and batch correction prior to statistical analyses.

#### Global signal normalization.

For each sample, the total signal intensity across all detected features was calculated. A scaling factor was then derived by dividing the median total signal across all samples by the total signal of each individual sample. Each metabolite intensity was multiplied by this scaling factor so that all samples had comparable total signal intensity across the dataset.

#### QC-based signal drift correction (LOESS).

To correct for signal drift occurring during long LC–MS acquisition sequences, a quality control (QC)-based LOESS correction was applied. QC samples injected periodically throughout the analytical run were used to estimate the temporal drift of each metabolite signal. For each feature, locally estimated scatterplot smoothing (LOESS) regression was fitted to the QC intensities as a function of injection order. The resulting smoothed drift curve was interpolated across the full run order, and metabolite intensities were corrected by dividing each observation by the estimated drift value and rescaling by the median signal. This procedure removes systematic signal changes attributable to instrument drift, estimated to be around 3% in the present study while preserving biological variability.

#### Batch correction.

Samples were acquired across multiple analytical batches during the study period. To remove batch-associated systematic variation, biological samples were batch corrected using the ComBat empirical Bayes method implemented in the neuroCombat Python package. Batch labels were extracted from sample metadata and incorporated as covariates in the model. Prior to batch correction, missing values were imputed using the per-feature median across biological samples. QC and control samples were excluded from batch correction to avoid introducing bias in the empirical estimation of batch parameters. The corrected metabolite matrix was subsequently used for downstream statistical analyses.

#### Relative abundance.

GraphPad Prism 10.0 was used for generating metabolites relative abundance plots (0 Gy vs 1Gy vs 4 Gy). Mann-Whitney and Kruskal-Wallis tests were applied as appropriate (significance: p < 0.05). Data were presented as mean ± standard error of the mean (SEM).

#### Discovery cohort.

Two complementary analytical objectives were pursued. First, supervised multi-omic integration (DIABLO) was used as an exploratory framework to identify candidate radiation-responsive features showing coordinated, dose-dependent separation across omics blocks within the discovery cohort dataset described above (see “Animal procedures” section, n = 6 per group). AUC values reported for DIABLO-derived features reflect within-cohort discrimination and are not intended as estimates of out-of-sample predictive performance. Second, to evaluate generalizability, candidate metabolite panels were independently assessed in a separate validation cohort not used during feature selection or model fitting.

#### Validation cohort.

An independent mouse cohort was used to evaluate the reproducibility of the metabolite signatures identified in the discovery dataset using the same experimental design: skin swab samples were collected from mice exposed to 0 Gy (n = 20), 1 Gy (n = 20), or 4 Gy (n = 20) of radiation at time points ranging from one to six days post-irradiation in a XRAD 320 irradiator (300 keV/10mA) fitted with a 0.5 mm Cu filter (HVL ≅ 1.7 mm Cu), at a dose rate of 1.3 Gy/min. These samples were not used during feature selection or model training. Samples were processed using the same metabolomic workflow and analytical pipeline applied to the discovery dataset. Candidate metabolites identified in the discovery analysis were evaluated in the validation cohort to assess reproducibility of metabolic perturbations. Replication was assessed by comparing fold-change patterns between cohorts and by evaluating classification performance using receiver operating characteristic (ROC) analysis with permutation testing.

#### Fold change analysis and validation of metabolite responses.

To evaluate the reproducibility of metabolite perturbations between discovery and validation cohorts, absolute fold changes, independently of direction, were calculated for each metabolite in both datasets. For each cohort, the absolute log2 fold change (log2FC) between irradiated (4 Gy) and control (0 Gy) groups was computed as:


$$\begin{array}{c} {\text{log}}_{\text{2}}FC={\text{log}}_{\text{2}}\left(\frac{{\text{mean intensity}}_{\text{4}Gy}}{{\text{mean intensity}}_{\text{0}Gy}}\right) \end{array}$$

The relationship between discovery and validation fold changes was evaluated using Pearson correlation analysis. Scatter plots were generated comparing fold changes obtained in the discovery cohort with those obtained in the validation cohort, and the Pearson correlation coefficient (R), coefficient of determination (R²), and associated p-value were reported to quantify the strength and statistical significance of the relationship.

## Results

### Molecular and microbial responses to radiation in coHSEs

**Fig 1:**
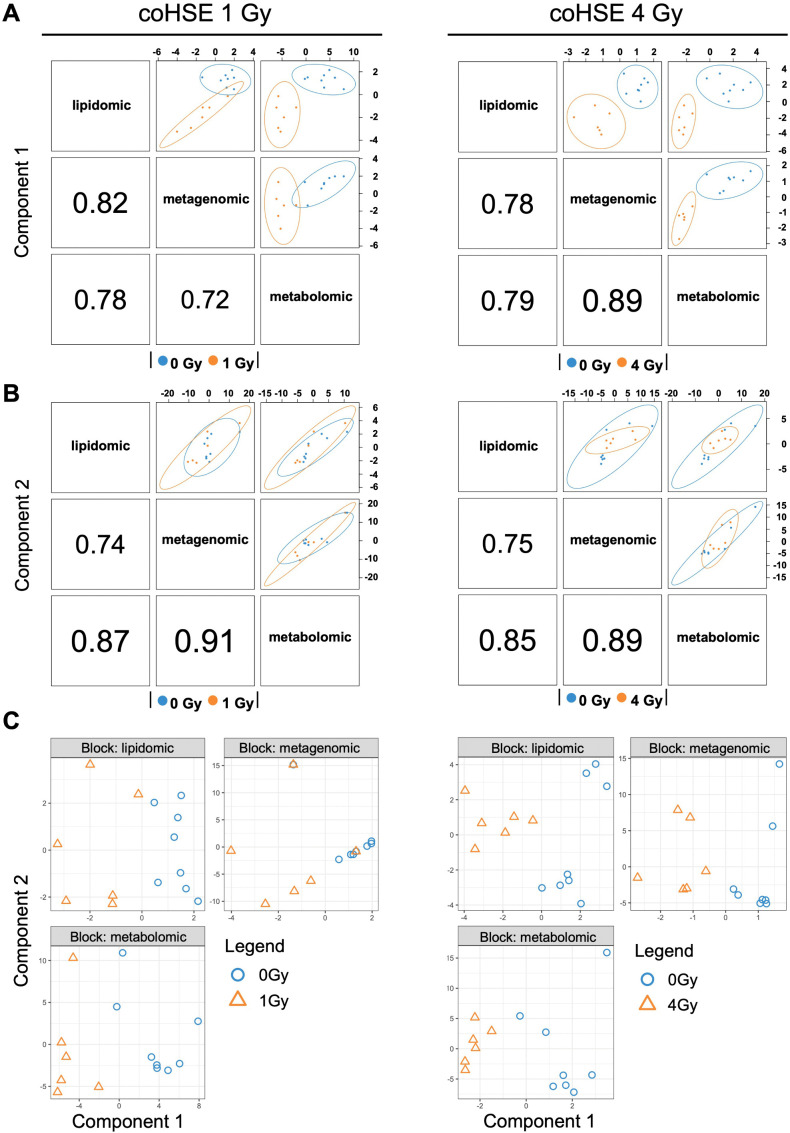
Performance of multi-omic integration and correlations. A-B) Diagnostic plots of DIABLO multiblock in component 1 and component 2 and C) DIABLO sPLS-DA plots in coHSE after radiation exposure using the top 100 correlating features in each block.

**Fig 2:**
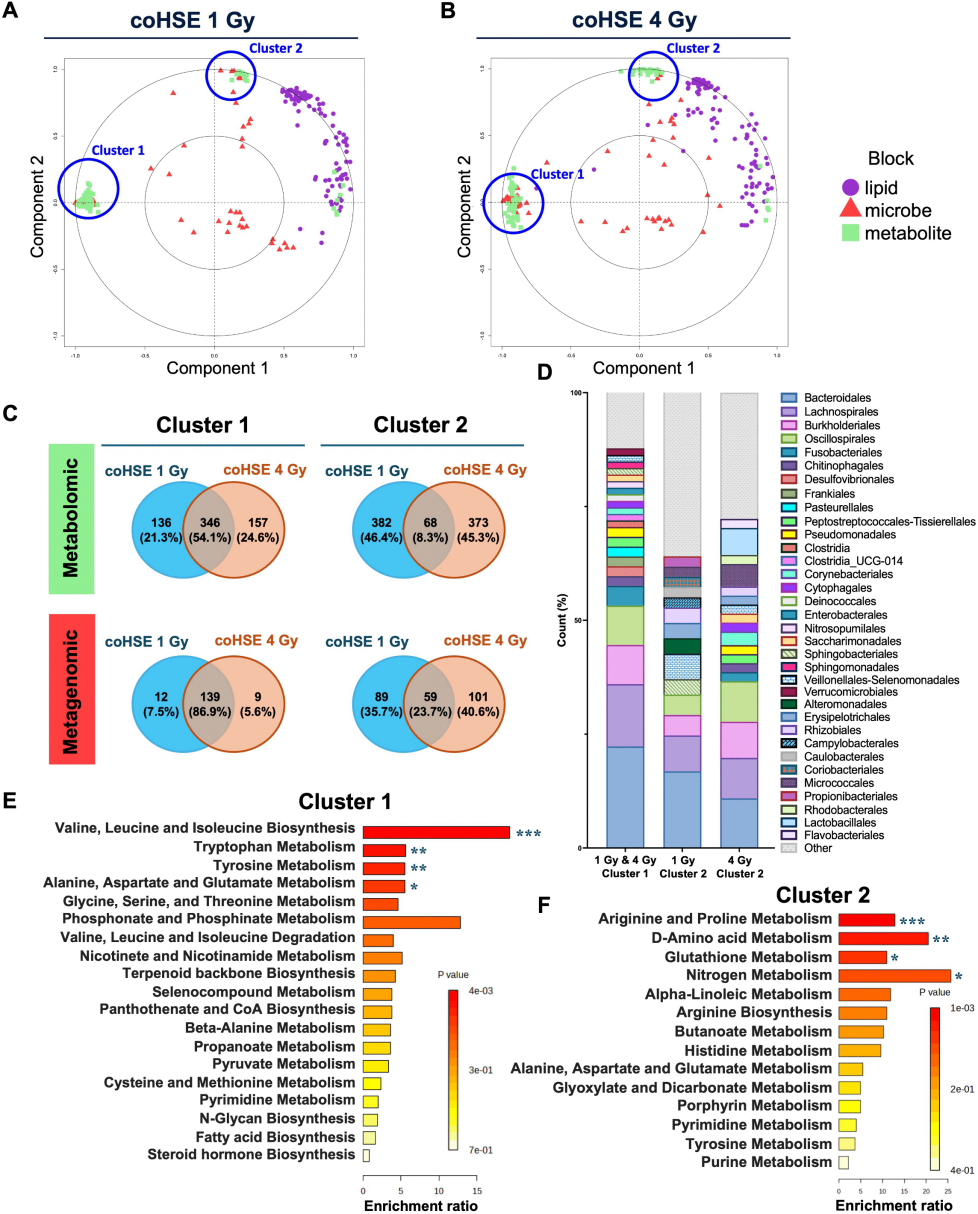
Multi-omic response to radiation exposure at 1 Gy and 4 Gy in coHSE. A-B) Correlation circleplots of 100 to features (DIABLO Multiblock sPLS-DA integration, C) Venn diagram showing common metabolomic and metagenomic features between 1 Gy and 4 Gy exposed coHSE incluster 1 and 2, D) top correlating taxa orders in clusters 1 and 2 at 1 Gy and 4 Gy, E) Over representation analysis (ORA) of top correlating putatively identified metabolites (0 > 0.5) common to 1 Gy and 4 Gy in cluster 1 using the KEGG library, and F) ORA of top correlating putatively identified metabolites (0 > 0.5) common to 1 Gy and 4 Gy in cluster 2 using the KEGG library, DIABLO: Data Integration Analysis for Biomarker Discovery using Latent Variable Approaches for Omics Studies. * p-value < 0.05, ** p-value < 0.01, *** p-value < 0.001.

To investigate the molecular and microbial profile response of the skin to radiation, we performed a Data Integration Analysis for Biomarker discovery using Latent variable approaches for Omics studies (DIABLO) multiblock sPLS-DA analysis integrating lipidomic, metabolomic, and metagenomic profiling of skin swab samples collected from coHSEs within the first week (pooled time points) post exposure to 1 Gy and 4 Gy of x-rays using the R package mixOmics [55]. As shown in Fig 1, correlations of latent variables across the 3 omics datasets were maximized in component 1 at both 1 Gy and 4 Gy, ranging from 0.72 to 0.89 (Fig 1A). Higher inter-block correlations were observed on component 2, particularly between the metabolomic and metagenomic blocks (r = 0.91 at 1 Gy; r = 0.89 at 4 Gy), though this component did not enhance group separation (Fig 1B). DIABLO sample plots using the 100 top inter-block correlating features demonstrated strong group separation across all three omics blocks, indicating that correlating changes in multi-omic profiles can discriminate between exposed and non-exposed groups (Fig 1C). Correlation circle plots from the multiblock sPLS-DA model (Fig 2A–B) revealed that two distinct microbiome-metabolome clusters (Clusters 1 and 2) are involved in the response of the skin to radiation at both 1 Gy and 4 Gy, while lipidomic features mainly correlated with metabolomic features. Feature-level overlap analysis of the clusters demonstrated a mixture of shared and dose-specific responses, among metabolomic and metagenomic signatures (Fig 2C). Cluster 1 was largely conserved between doses, with 346 metabolomic and 139 metagenomic features shared between 1 Gy and 4 Gy, accounting for 54.1% and 86.9% of the cluster composition respectively. Conversely, Cluster 2 exhibited dose-specific divergence: 68 metabolomic features (8.3%) and 59 taxa (23.7%) were commonly found in 1 Gy and 4 Gy, while 382 (46.4%) metabolomic features and 89 (35.7%) taxa were exclusive to 1 Gy, and 373 (45.3%) metabolomic features and 101 (40.6%) taxa were exclusive to 4 Gy. These results suggest the presence of both conserved responses and dose-dependent radiation responses in the skin. Taxonomic profiling of cluster 1 and 2 showed that both 1 Gy and 4 Gy microbial signatures involved *Bacteroidales*, *Lachnospirales*, *Burkholderiales*, and *Oscillospirales* orders (Fig 2D). Conversely, *Lactobacillales* taxa from cluster 2 were found exclusively at 4 Gy, while *Propionibacteriale*, *Coriobacteriales, Campylobacterales*, and *Caulobacterales* were exclusive to 1 Gy.

To gain mechanistic insight into the biochemical pathways contributing to cluster separation, we performed over-representation analysis (ORA) of the top correlating putatively identified metabolites found in common at 1 Gy and 4 Gy using the Kyoto Encyclopedia of Genes and Genomes (KEGG) library. In Cluster 1 (Fig 2E), common enriched pathways included valine, leucine, and isoleucine biosynthesis, tryptophan and tyrosine metabolism, and alanine, aspartate and glutamate metabolism. These signatures suggest a metabolic shift toward amino acid turnover following radiation exposure. In Cluster 2 (Fig 2F), the metabolite profile pointed toward elevated oxidative and inflammatory signaling, with enrichment in arginine and proline metabolism, D-amino acid metabolism, nitrogen metabolism, glutathione metabolism, and mitochondrial-related pathways such as butanoate, pyrimidine, and purine metabolism. KEGG pathway enrichment on the exclusive metabolite subsets from Cluster 2 of each dose group (S1 Fig) showed that the 382 metabolites unique to the 1 Gy group were enriched in arachidonic acid metabolism, linoleic acid metabolism, histidine metabolism, and amino acid biosynthesis and degradation (e.g., tryptophan, D-amino acids). These pathways suggest activation of lipid inflammatory mediators and oxidative stress responses. By contrast, the 373 metabolites exclusive to the 4 Gy group were associated with broader mitochondrial function and lipid perturbations, including ubiquinone metabolism, pantothenate and CoA metabolism, steroid hormone biosynthesis, riboflavin metabolism, pyruvate metabolism, taurine and hypotaurine metabolism, and glycolysis/gluconeogenesis. Enrichment of the citrate cycle (TCA), selenocompound metabolism, and primary bile acid biosynthesis point to widespread metabolic dysfunction and energy imbalance at higher doses.

Phenylalanine, tyrosine, and tryptophan were significantly reduced at 4 Gy, in line with enriched catabolic pathways observed in Cluster 1 (S2 Fig). Similarly, the depletion of xanthine, hippuric acid, and glutarylcarnitine reflected altered purine metabolism and impaired mitochondrial function. Although NMF-related amino-acid like proline, and urocanic acid (UCA), pyrrolidone carboxylic (PCA) acid and lactic acid remained stable, arginine and glutamic acid were drastically depleted in exposed groups while alanine were increased at 4 Gy, suggesting potential alterations in the epidermal barrier properties after radiation exposure (S3 Fig).

We performed a lipid reaction network analysis to predict lipid class enrichment based on lipid reaction z-scores using the online software Lipidmaps BioPan (S4 Fig). At 1 Gy the lipid networked was dominated by an enrichment in diacylglycerols (DG) from suppressed conversion in monoacylglycerols (MG) Gy (z-scores = −1.787), complemented by an enrichment in phosphatidylserines (PS) from activated conversions of phosphatidic acid (PA) Gy (z-scores = 1.925) and phosphatidylcholine (PC) (z-scores = 1.754) also observed at 4 Gy (z-scores = 1.742). This suggests a pronounced role in phospholipid remodeling and/or potential upregulation of membrane biogenesis after radiation exposure.

### Multi-omic integration uncovers distinctive skin response to 1 Gy and 4 Gy radiation in mice

**Fig 3:**
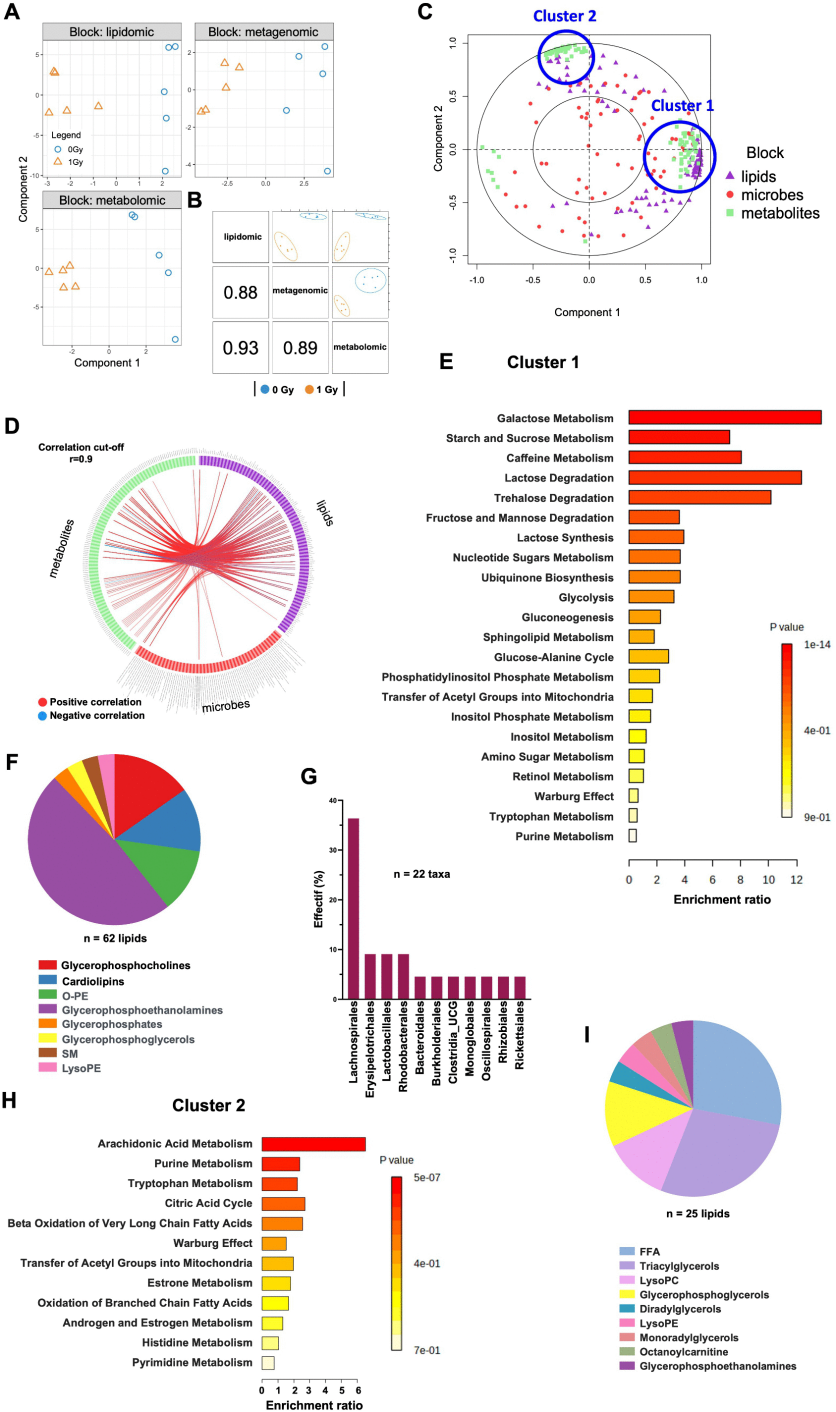
Molecular and microbial response to radiation exposure at 1 Gy in the mouse skin. A) Sample plots of DIABLO Multiblock sPLS-DA integration (lipidomic, metagenomic, metabolomic) of 100 top features in the skin of mice at 1 Gy compare to 0 Gy B) Diagnostic plot of DIABLO Multiblock showing correlation coefficients between blocks (lipidomic vs metagenomic = 0.88, metagenomic vs metabolomic = 0.89, lipidomic vs metabolomic = 0.93) and 95% confidence ellipses using top 100 features in the first dimension (component 1) C) Correlation circle plot of top 100 discriminating features D) Circos plot showing correlations between the top 100 features in lipidomic, metabolomic, and metagenomic E) Over representation analysis (ORA) of top correlating putatively identified metabolites (correlation >0.5) in cluster 1 using the SMPDB, F) ORA of top correlating lipids in cluster 1, G) ORA of top correlating taxa (order) in cluster 1, H) ORA) of top correlating putatively identified metabolites (correlation >0.5) in cluster 2 using the SMPDB, and I) ORA of top correlating lipids in cluster 2. DIABLO: Data Integration Analysis for Biomarker Discovery using Latent Variable Approaches for Omics Studies.

In mice, DIABLO sPLS-DA analysis of skin swab samples collected within the first week post- exposure to x-rays (pooled time points) also revealed clear separation between irradiated (1 Gy) and control (0 Gy) groups (Fig 3A). Inter-block correlations were strong in the first component (lipidomic vs. metagenomic = 0.88; lipidomic vs. metabolomic = 0.93; metagenomic vs. metabolomic = 0.89; Fig 3B) supporting robust multi-layered connectivity. However, group separation was poor in the second component (S5 Fig) and variables in this dimension were excluded from biomarker identification (Fig 5–8). Two major feature clusters were also observed in the correlation circle plot (Fig 3C), with cluster 1 involving all three blocks and cluster 2 comprising exclusively metabolomic and lipidomic features. Circos plot mapping (r ≥ 0.9) confirmed strong inter-block associations, particularly between metabolites and lipids (Fig 3D). Enrichment analysis of cluster 1 metabolomic features (correlation > 0.5) showed significant activation of carbohydrate-related processes (e.g., galactose, starch, lactose metabolism), and glycolytic flux (glycolysis, gluconeogenesis, glucose-alanine cycle, Warburg effect) (Fig 3E). Lipids in this cluster were mainly dominated by glycerophosphoethanolamines (~50%), followed by O-PE, cardiolipins (CL), and glycerophosphocholines (PC) (Fig 3F), while microbial populations were mainly dominated by *Lachnospirales* (~40%) (Fig 3G and S3 Table). Other taxa in cluster 1 included *Lactobacillales*, *Burkholderiales, Bacteroidales* and *Oscillospirales*. In contrast, cluster 2 metabolomic features were enriched in lipid pathways such as arachidonic acid metabolism and fatty acid β-oxidation, which are associated with oxidative stress and inflammation, as well as nucleic acid metabolism (e.g., pyrimidine and purine metabolism). Enrichment in TCA cycle metabolism also indicate a potential effect of this cluster in mitochondrial function (Fig 3H). Corresponding lipids included free fatty acids (FFA), triacylglycerols (TG), and LysoPCs (Fig 3I), also suggestive of inflammatory signaling, lipid mobilization, and barrier remodeling. Lipid reaction network analysis (S6 Fig) further detailed the directionality of lipid changes following radiation exposure. Consistent with coHSE lipid network at 1 Gy, DG nodes were also highly enriched from both suppressed and activated reactions with TG and MG respectively in the mouse skin at 1 Gy, with Z-scores up to +2.66, indicating a strong dynamic between these lipid classes.

**Fig 4:**
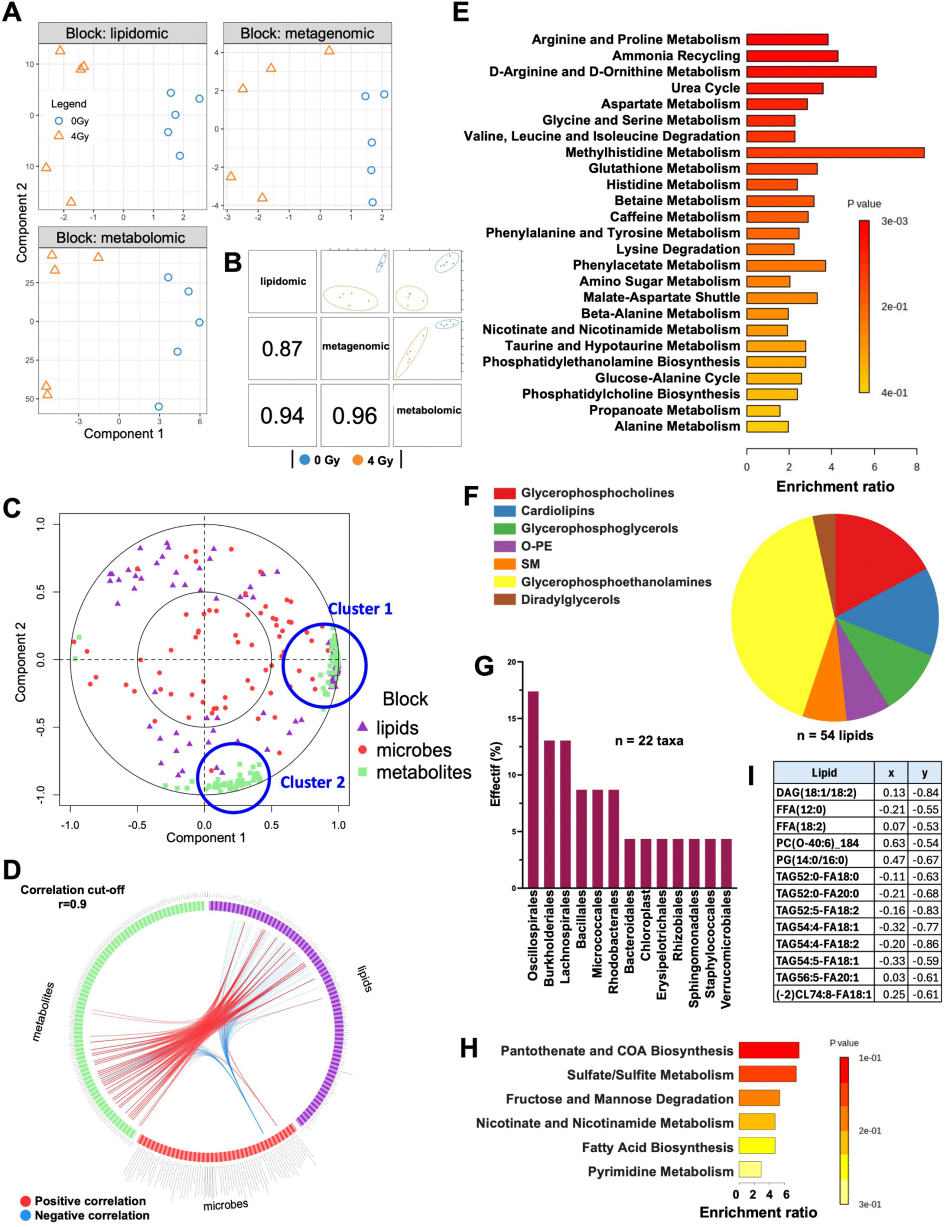
Molecular and microbial response to radiation exposure at 4 Gy in the mouse skin. A) Sample plots of DIABLO Multiblock sPLS-DA (lipidomic, metagenomic, metabolomic) of 100 top features in the skin of mice at 4 Gy compare to 0 Gy B)) Diagnostic plot of DIABLO Multiblock showing correlation coefficients between blocks (lipidomic vs metagenomic = 0.87, metagenomic vs metabolomic = 0.96, lipidomic vs metabolomic = 0.94) and 95% confidence ellipses using top 100 features in the first dimension (component 1) C) Correlation circle plot of top 100 features D) Circos plot showing correlations between the top 100 features in lipidomic, metabolomic, and metagenomic E) Over representation analysis (ORA) of top correlating putatively identified metabolites (correlation>0.5) in cluster 1 using the SMPDB, F) ORA of top correlating lipids in cluster 1, G) ORA of top correlating taxa (order) in cluster 1, H) ORA) of top correlating putatively identified metabolites (correlation >0.5) in cluster 2 using the SMPDB, and I) Top correlating lipids in cluster 2. DIABLO: Data Integration Analysis for Biomarker Discovery using Latent Variable Approaches for Omics Studies. CL denotes cardiolipin species, reported as total carbon number and double bonds (e.g., CL66:3). Instrument-specific annotations (e.g., “(-2)”) correspond to feature identifiers used in lipidomics analysis.

At 4 Gy, irradiated samples again separated clearly from controls across the three blocks (Fig 4A), and cross-block correlations were further enhanced compared to 1 Gy (lipidomic vs. metagenomic = 0.87; metagenomic vs. metabolomic = 0.96; lipidomic vs. metabolomic = 0.94) (Fig 4B) (S5 Fig). Correlation circle plots again revealed two distinct feature clusters (Fig 4C), with strong positive correlations linking metabolomic and microbial variables (Fig 4D). Follow-up enrichment analysis of most correlating features (correlation > 0.5) indicated that metabolomic features in cluster 1 were predominantly associated with commonly observed radiation-induced pathways including both glucogenic and ketogenic amino acid metabolism including key component of skin NMF (e.g., arginine/proline, aspartate, glycine/serine, histidine, phenylalanine/tyrosine, taurine, alanine, and lysine metabolism and glutathione), nitrogen disposal (ammonia recycling, D-arginine/D-ornithine metabolism, urea cycle), cellular redox regulation (e.g., glutathione, nicotinate and nicotinamide metabolism, malate-aspartate shuttle) (Fig 4E), and phospholipid anabolism (phosphatidylethanolamine and phosphatidylcholine biosynthesis). Consistently, Cluster 1 lipids were also enriched in PE, PC as well as CL (Fig 4F), suggesting a critical role of phospholipids in the skin response to radiation. CLs especially, are structurally unique phospholipids synthesized exclusively in mitochondria, indicating a potential disruption of the mitochondrial function at both doses. Taxa in cluster 1, included *Oscillospirales*, *Burkholderiales, Lachnospirales* (Fig 4G, S3 Table). However, unlike at 1 Gy, *Lactobacillales* were absent, while *Bacillales* and *Micrococcales* were newly prominent, suggesting dose-dependent microbiome restructuring. In cluster 2, enriched pathways involved in Coenzyme A and fatty acid biosynthesis, sulfate metabolism, and pyrimidine metabolism, suggesting a broader regulatory role of this cluster spanning carbohydrates, fats, and proteins metabolism (Fig 4H). As in 1 Gy, microbial features were mostly absent from cluster 2, and dominant lipids in this cluster at 4 Gy also included FFA and TG species, as well as DG (Fig 4I), consistent with enhanced anabolic lipid flux and oxidative stress response. Contrary to coHSE at the same dose, the DG node remained central in lipid flux (z-scores = +1.708), while PA exhibited stronger positive enrichment due to suppressed reactions with LPA (z-scores = +1.882), and little reactions with PS (z-scores = −0.011) contrary to 4 Gy coHSE groups (S6 Fig).

In summary, integrative multi-omic profiling of mouse skin swabs uncovered distinct and dose-specific signatures in response to ionizing radiation. 1 Gy radiation exposure was primarily associated with the activation of carbohydrate metabolism pathways, while concurrent changes in the abundance of microbial taxa such as Lachnospiraceae were observed. In contrast, 4 Gy correlated mainly with perturbation in amino acid metabolism including components of the NMF, detoxification and redox regulatory pathways such as glutathione metabolism and the urea cycle, changes that were mainly correlated to *Oscillospirales*, *Burkholderiales,* and *Lachnospirales* families. Additionally, these responses are orchestrated along two principal axes: (1) a metabolome-microbiome axis principally involving *Lachnospirales, Oscillospirales*, and *Burkholderiales* taxa (Cluster 1) and defined by phospholipid alterations, energy production shifts and disruption of pathways involved in the skin barrier function, and (2) a metabolome-lipidome axis (Cluster 2) marked by inflammatory lipidic signatures, potentially affecting all biomolecules classes, and a relative decoupling from microbial influence. Altogether, these observations underscore the capacity of integrated omics to resolve the temporal and dose-dependent complexity of radiation responses in skin and reinforces the translational potential of dermal-based biodosimetry.

### Skin microbial and lipid biomarkers distinguish radiation exposure in mice

**Fig 5:**
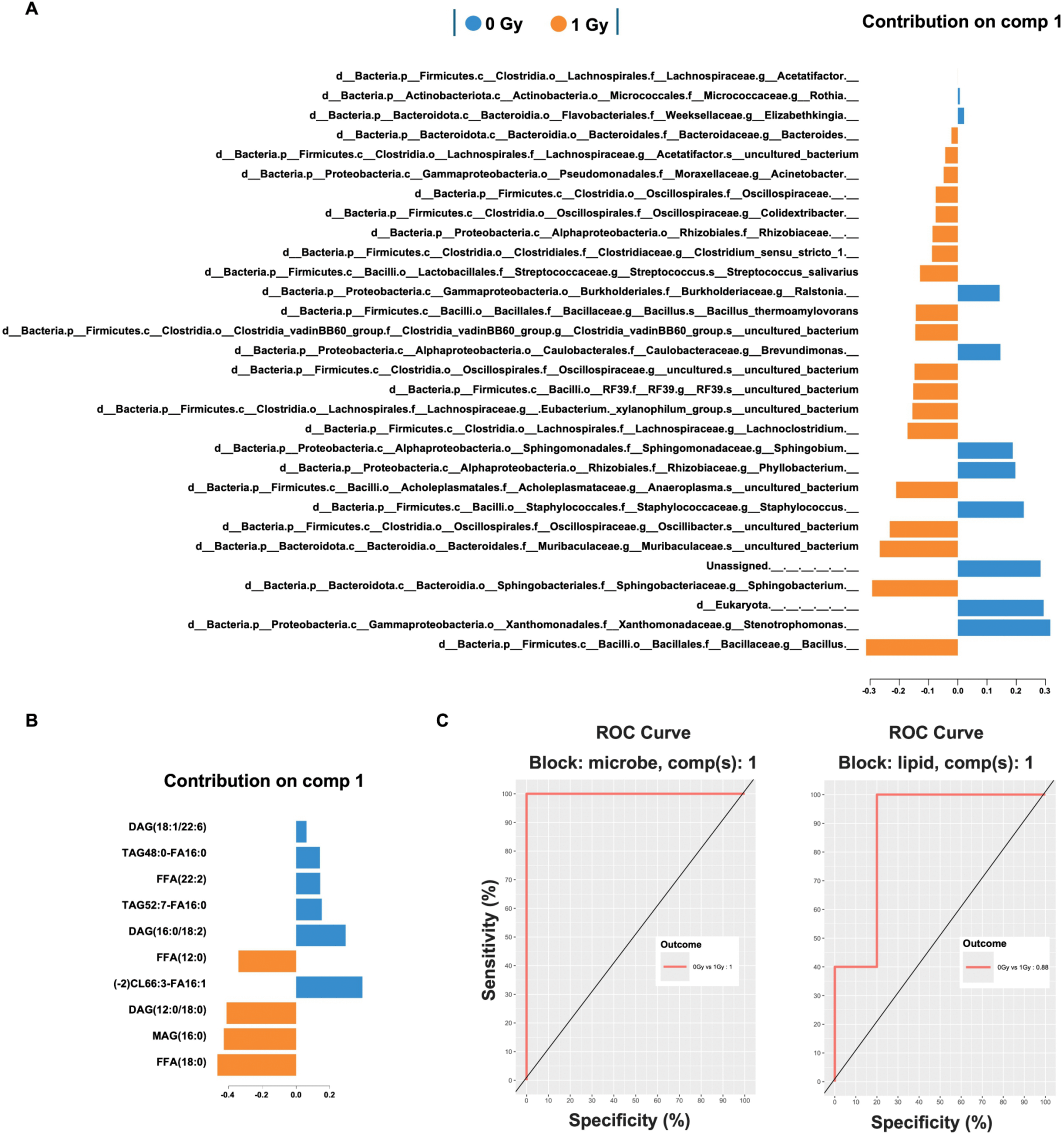
Metagenomic and lipidomic signatures of radiation exposure at 1 Gy in the mouse skin. A and B) Loading plot for metagenomic and lipidomic after DIABLO Multiblock sPLS-DA integration at 1Gy in mouse skin. The most important variables (according to the absolute value of their coefficients) are ordered from bottom to top. C) ROC curve with most important variables in metagenomic and lipidomic. CL denotes cardiolipin species, reported as total carbon number and double bonds (e.g., CL66:3). Instrument-specific annotations (e.g., “(-2)”) correspond to feature identifiers used in lipidomics analysis. Lipidomic AUC: 95% CI 0.302–1.000, 100 permutation p = 0.248, metagenomic AUC: 95% CI 0.167–1.000, 100 permutation p = 0.356.

To identify key microbial taxa and lipid species that can serve as candidate biomarkers for radiation exposure in the skin, we extracted most discriminative features driving classification between irradiated (1 Gy) and control (0 Gy) samples, derived from DIABLO multiblock sPLS-DA integration of metagenomic and lipidomic datasets as shown in loading plots in Fig 5. For each block, variables contributing most to group separation along components 1 were ranked based on the absolute value of their coefficients. In the metagenomic block (Fig 5A), component 1 revealed a set of 30 bacterial taxa and 10 lipids enriched in either 1 Gy or 0 Gy groups. Notably, *Bacillaceae*, *Sphingobacteriaceae*, *Muribaculacae*, *Streptococcaceae*, *Oscillospiraceae*, and *Lachnospiraceae* families were highly weighted in the 1 Gy group, the two later being particularly recurrent, whereas taxa such as *Xanthomonadaceae*, *Staphylococcaceae*, *Caulobacteriaceae,* and *Bulkholderiaceae* were associated with control samples. In the lipidomic block (Fig 5B), a general observation is that top discriminating lipids increased in controls included polyunsaturated fatty acids (PUFA) such as DAG(18:1/22:6), FFA(22:2), TAG(52:7)-FA(16:0), and (−2)Cl66:3-FA(16:1), whereas top discriminating lipids increased in the irradiated group included saturated FA only, such as the FFA(18:0), a radiation-associated FFAs, MAG(16:0), DAG(12:0/18:0), and FFA (12:0). We constructed ROC curves for both the metagenomic and lipidomic blocks to evaluate classification performance using these most predictive features (Fig 5C). High AUC values in both blocks (microbe = 1, lipid = 0.88) indicated strong within-sample separation of the selected features for distinguishing 1 Gy exposure from control samples. Permutation analysis were not statistically significant (lipidomic permutation p = 0.248, metagenomic permutation p = 0.356) indicating possible overfitting of the identified signature in the discovery cohort and the need for validation in an independent cohort.

**Fig 6:**
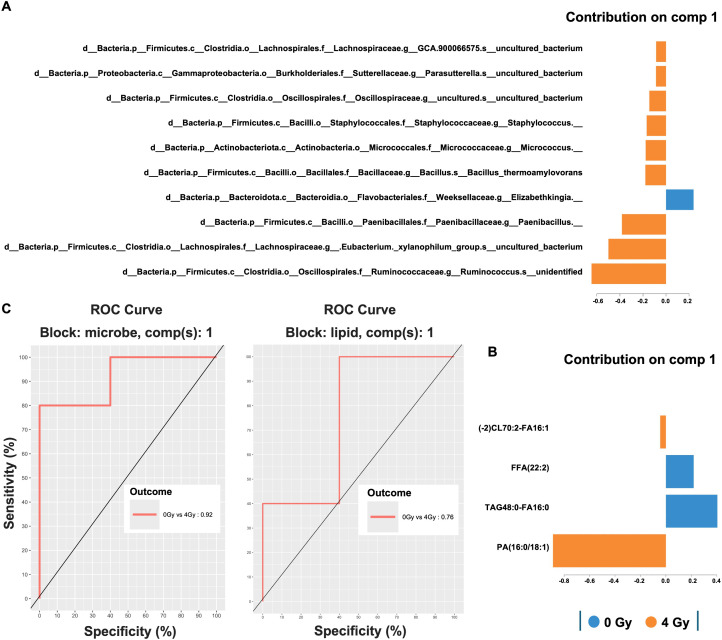
Metagenomic and lipidomic signatures of radiation exposure at 4 Gy in the mouse skin. A and B) Loading plot for metagenomic and lipidomic after DIABLO Multiblock sPLS-DA integration at 1 4 Gy in mouse skin. The most important variables (according to the absolute value of their coefficients) are ordered from bottom to top. C) ROC curve with most important variables in metagenomic and lipidomic. CL denotes cardiolipin species, reported as total carbon number and double bonds (e.g., CL66:3). Instrument-specific annotations (e.g., “(-2)”) correspond to feature identifiers used in lipidomics analysis. Lipidomic AUC: 95% CI 0.490–1.000, 100 permutation p = 0.158, metagenomic AUC: 95% CI 0.142–1.000, 100 permutation p = 0.337.

At 4 Gy, radiation-enriched taxa included again *Lachnospiraceae, Bacillaceae* and *Oscillospiraceae,* as well as *Ruminococcaceae, Micrococcaceae*, and *Staphylococcaceae*, while the 0 Gy group included only the *Weeksellaceae* family (Fig 6A). In the lipidomic block (Fig 6B), the most discriminative lipids included PA(16:0/18:1) and (−2)Cl70:2-FA(16:1), which were elevated in the 4 Gy group. Interestingly, FFA(22:2) and TAG(48:0)-FA(16:0) were discriminative for both 1 Gy vs 0 Gy and 4 Gy vs 0 Gy making them potential biomarkers of dose-dependent radiation exposure. Receiver operating characteristic (ROC) curve analysis confirmed that 10 microbial and 6 lipidomic top-ranked features provided strong classification performance distinguishing 0 Gy and 4 Gy groups with an area under the curve (AUC) of 0.92 and 0.76 respectively (Fig 6C), supporting their candidacy for further evaluation as dose-specific dermal biomarkers. Together, these results define a distinctive metagenomic and lipidomic signature of 4 Gy radiation, characterized by microbial dysbiosis and lipid remodeling in the mouse skin. Permutation analysis again, were not statistically significant confirming the need for validation in an independent cohort (lipidomic permutation p = 0.158, metagenomic permutation p = 0.337).

### Metabolomic signatures differentiate 1 Gy and 4 Gy radiation exposure and reveal diagnostic potential

**Fig 7:**
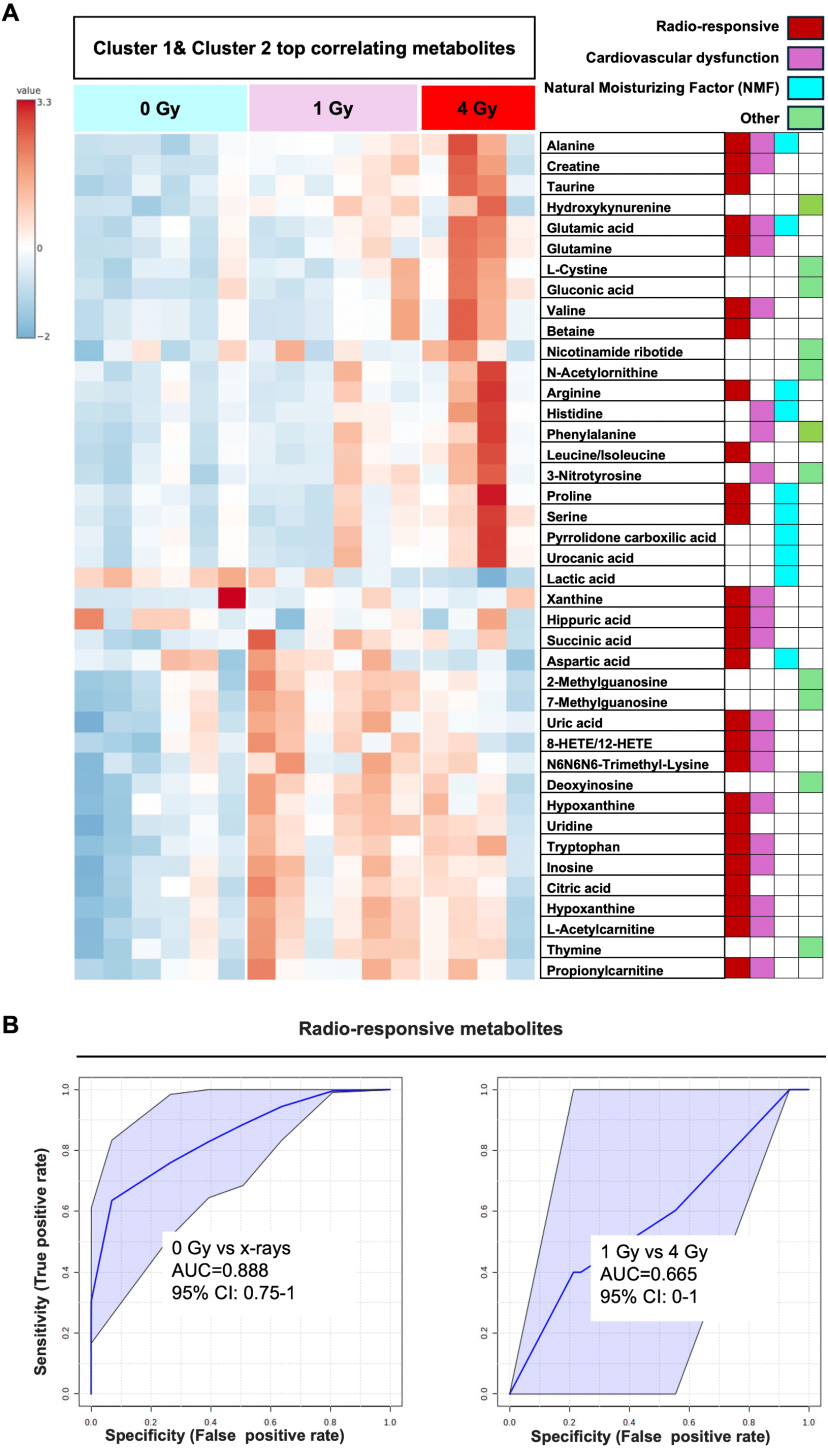
Metabolomic signature of radiation exposure in the mouse skin. A) ANOVA heatmap of most correlating putatively identified metabolites (sPLS-DA), among which 26 already reported radio-responsive metabolites, 10 components of the natural moisturizing factor (NMF) of the skin, 20 cardiovascular health markers, and 11 new radiation exposures candidate biomarkers (others), B) Receiver operating characteristic (ROC) curve analysis of non-exposed (0 Gy) vs exposed (1 Gy and 4 Gy)and 1 Gy vs 4 Gy using the most correlating putatively identified metabolite panel (Random forest).

The spectral abundance of most strongly correlated putatively identified metabolites in mice across all radiation conditions using sPLS-DA and ANOVA are shown in Fig 7. The heatmap displays 52 discriminative metabolites across 0 Gy, 1 Gy, and 4 Gy, including 26 previously reported radio-responsive metabolites, 10 components of the skin’s natural moisturizing factor (NMF), 20 cardiovascular health–associated metabolites, and 11 new candidate radiation biomarkers (Fig 7A). Several amino acids (e.g., taurine, arginine, histidine), NMF components (e.g., urea, glutamic acid, serine), and oxidative stress–linked metabolites (e.g., hydroxykynurenine, uric acid, 8-HETE) showed clear dose-dependent modulation from 0 Gy to 4 Gy. Most of the NMF-related metabolites showed a consistent progressive upregulation with dose, suggesting disruption of the dermal barrier. Clustering analysis revealed two major metabolite groups with divergent radiation dose responsiveness.

To assess the classification performance of these features, we analyzed ROC curves generated using a random forest classifier (Fig 7B). The full metabolite panel showed strong discrimination of the discovery dataset between non-irradiated and irradiated samples (0 Gy vs. x-rays, AUC = 0.888; 95% CI = 0.75–1), but performance dropped for dose discrimination between 1 Gy and 4 Gy (AUC = 0.665; 95% CI = 0–1), indicating greater inter-individual variability and reduced resolution at higher doses. Supplementary Fig 7 further evaluates the diagnostic value of biologically annotated metabolite subsets. The NMF panel demonstrated excellent classification of exposure (0 Gy vs. 4 Gy, AUC = 1; 95% CI = 1–1), but lower reliability for 4 Gy vs. 1 Gy (AUC = 0.84; 95% CI = 0.333–1). Similarly, the “other” candidate biomarker panel performed well for distinguishing 1 Gy vs. 0 Gy (AUC = 0.965; 95% CI = 0.75–1) but showed a broader CI for 4 Gy vs. 1 Gy (AUC = 0.89; 95% CI = 0.333–1), confirming limited resolution between moderate and high-dose exposures using static metabolite profiles.

### Dose discrimination using a refined metabolite panel in mouse skin

**Fig 8:**
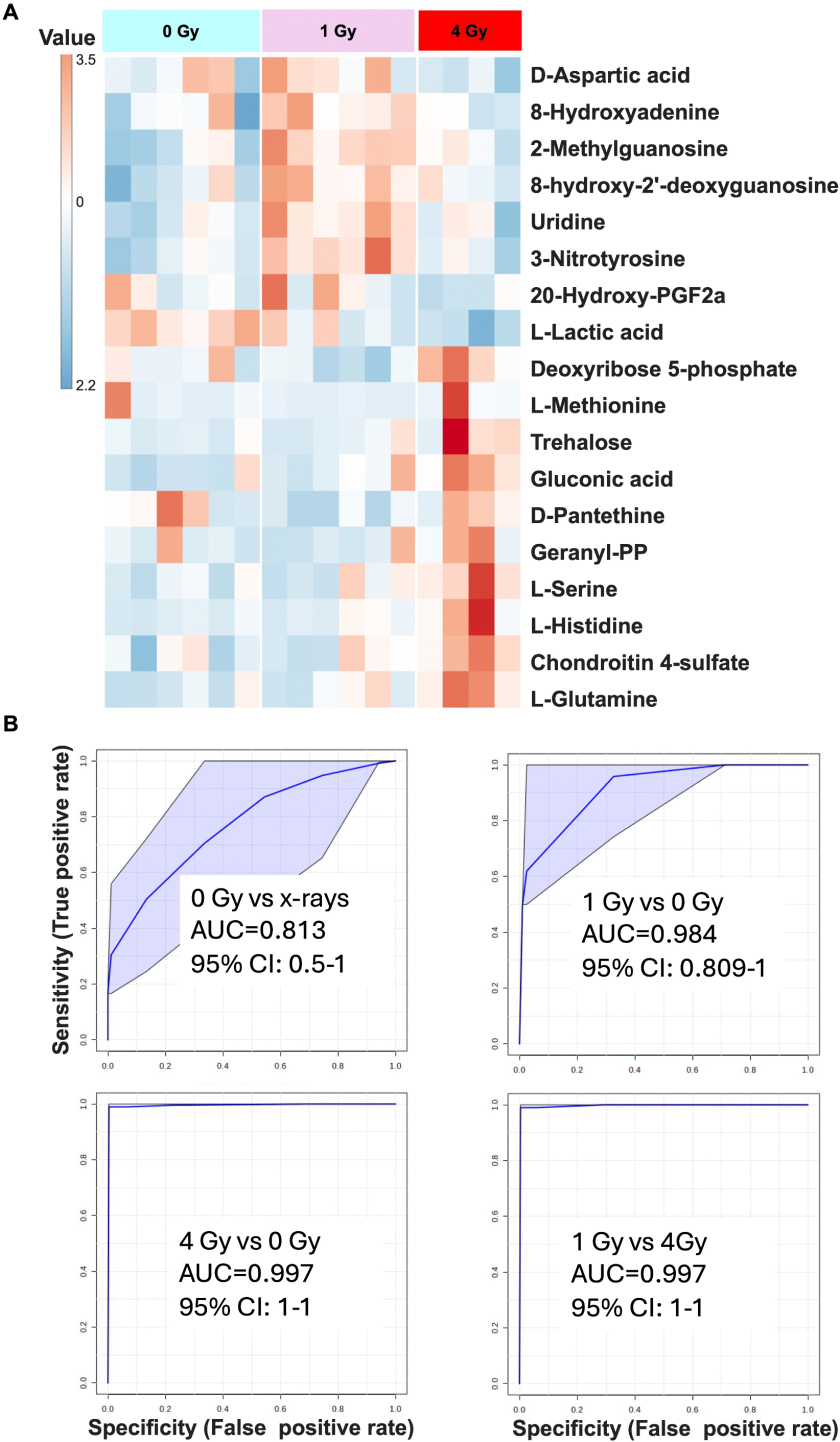
Dose discrimination with metabolomic features in the mouse skin. A) ANOVA heatmap of 18 most predictive putatively identified metabolites (Random forest 1Gy vs 4Gy AUC > 0.8) B) Receiver operating characteristic (ROC) curve analysis of non-exposed (0 Gy) vs exposed (1 Gy and 4 Gy), 0 Gy vs 1 Gy, 0 Gy vs 4 Gy, and 1 Gy vs 4 Gy using the 18 most predictive putatively identified metabolite panel (Random forest).

To enhance the resolution between moderate and high radiation doses, we selected the 18 most predictive putatively identified metabolites based on random forest classification performance (AUC > 0.8 for 1 Gy vs. 4 Gy) and visualized their patterns of abundance change using a heatmap (Fig 8A). This refined panel includes radiation-associated metabolites such as 3-nitrotyrosine, 8-hydroxy-2’-deoxyguanosine, 20-hydroxy-PGF2α, L-histidine, and D-aspartic acid, many of which are implicated in oxidative stress, DNA damage, and inflammation. Heatmap clustering confirmed clear dose-dependent separation across 0 Gy, 1 Gy, and 4 Gy conditions, with several features strongly upregulated at 4 Gy. ROC curve analyses confirmed the high discriminatory power of this panel within the discovery dataset across all pairwise comparisons (Fig 8B). Within the discovery cohort, the refined panel showed strong dose separation (AUC = 0.997), though this estimate reflects within-sample discrimination prior to independent validation. Other comparisons also showed strong performance: 0 Gy vs. 4 Gy (AUC = 0.997), 1 Gy vs. 0 Gy (AUC = 0.984; 95% CI = 0.809–1), and 0 Gy vs. all x-rays (AUC = 0.813; 95% CI = 0.5–1).

### Generalization and performance test of the identified metabolite panels in an independent mouse validation cohort

**Fig 9:**
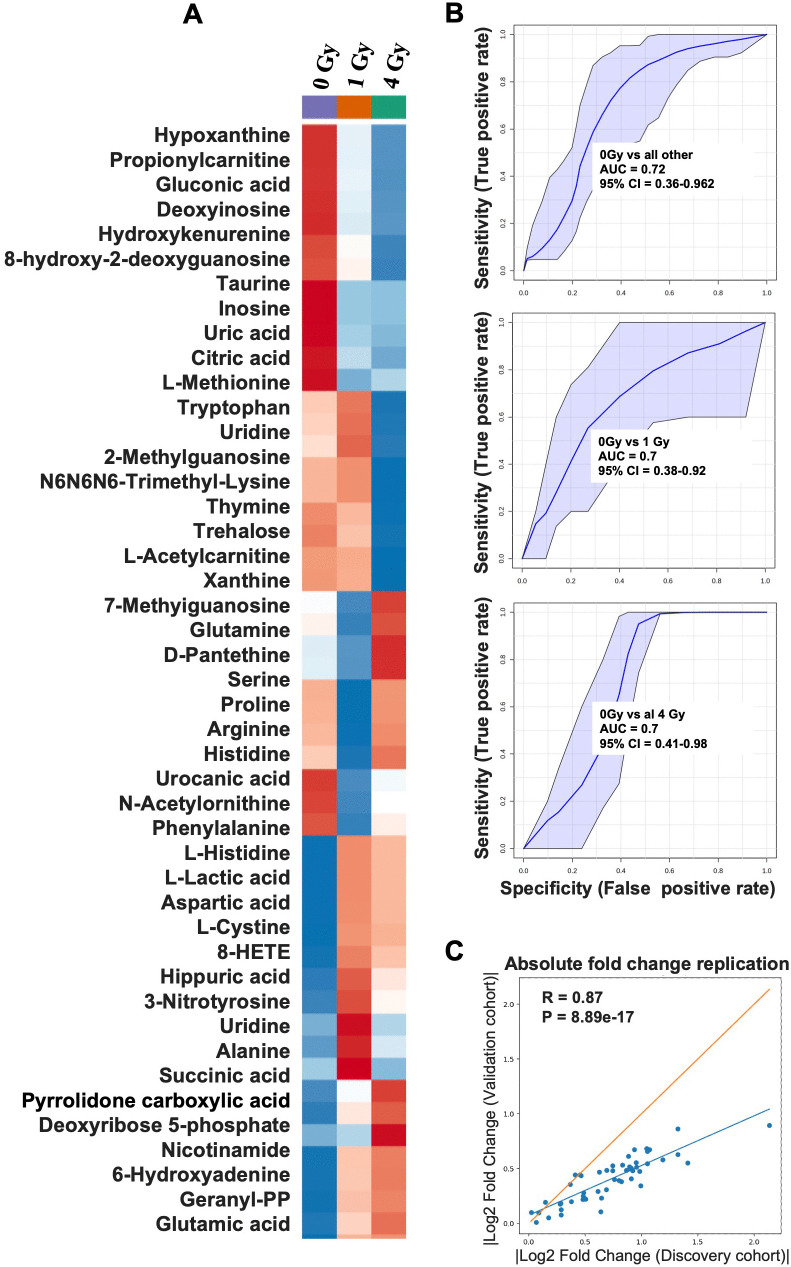
Independent validation of radiation-responsive skin metabolite panels and dose performance evaluation. A) Heatmap showing relative abundance of candidate metabolites across the validation cohort for 0 Gy, 1 Gy, and 4 Gy exposure groups, demonstrating conserved dose-dependent metabolic patterns. B) Receiver operating characteristic (ROC) analyses evaluating classification performance of the metabolite panel for distinguishing non-exposed from exposed samples and for dose discrimination, with permutation testing to assess statistical significance. C) Replication analysis comparing metabolite fold changes between discovery and validation datasets. Absolute fold-change effects were strongly correlated (R = 0.87), indicating reproducible magnitude of metabolic alterations across cohorts.

Because discovery-phase AUCs are expected to overestimate generalizable performance due to feature selection on the same dataset, we evaluated these candidate signatures in an independent mouse cohort. Independent validation in a separate mouse cohort reproduced consistent metabolic perturbations (fig 9 A). Although this metabolite signature showed moderate classification performance (AUC ≈ 0.70–0.72, Fig 9 B), absolute fold-change effects were strongly correlated (R = 0.87, p = 8.89 10^-17^, Fig 9C), indicating reproducible magnitude of metabolite alterations across cohorts.

## Discussion

### Summary of key findings

With the increasing use of radiation-producing technologies, the risk of nuclear exposure, and human expansion into space, there is a growing need for non-invasive biomarkers enabling rapid biodosimetry and longitudinal monitoring. As the outermost and highly responsive organ, the skin represents a promising matrix for exposure assessment. Here, integrative multi-omic profiling of skin swabs using DIABLO-based sPLS-DA successfully discriminated radiation dose and identified conserved biological signatures across experimental models, supporting the feasibility of skin-based biodosimetry. Importantly, independent validation in a separate mouse cohort demonstrated reproducible metabolite perturbations with strong concordance in effect size despite only moderate classification performance, supporting the robustness of the identified signatures while highlighting the need for further optimization for predictive applications.

Across both coHSE and mouse skin, radiation responses were driven by coordinated covariance among lipidomic and metabolomic pathways, including DAG-centered lipid networks, free fatty acids and glycerophospholipids, nitrogen and energy metabolism, and components of the natural moisturizing factor (NMF). These metabolic signatures were accompanied by consistent microbial shifts involving *Lachnospirales*, *Lactobacillales*, *Burkholderiales*, and *Oscillospirales*, suggesting that radiation exposure induces an integrated host–microbiome response detectable in skin swabs.

In the coHSE model, multi-omic integration revealed two distinct response clusters. The first represented conserved radiation responses across doses, characterized by changes in energy metabolism and NMF-related amino acid pathways, suggesting a generalized regulatory component of the skin radiation response. In contrast, the second cluster exhibited dose-dependent perturbations, with 1 Gy associated with inflammatory and oxidative lipid signaling (e.g., arachidonic and histidine metabolism), whereas 4 Gy induced broader mitochondrial and energetic disruptions, including glycolysis, the TCA cycle, and CoA biosynthesis. Importantly, dose-dependent alterations in metabolites associated with NMF synthesis suggest potential impairment of epidermal barrier homeostasis, providing candidate markers exploitable for skin-based biodosimetry.

Consistent patterns were observed in mouse skin, where 1 Gy primarily affected carbohydrate and lipid metabolism, while 4 Gy produced broader amino acid, redox, and nitrogen metabolism changes, accompanied by lipid remodeling. Microbial shifts also displayed dose-specific features, *with Lactobacillales* enriched at 1 Gy and *Bacillales/Micrococcales* emerging at 4 Gy, indicating structured microbial reorganization following radiation exposure.

### Microbial-molecular dynamics

Members of the *Lachnospiraceae*, *Burkholderiaceae*, *Oscillospirales*, and *Lactobacillales* taxa were among the most significantly altered microbial groups in both coHSE and mouse skin following radiation exposure, consistent with prior reports of radiation-responsive bacteria in terrestrial and spaceflight contexts [35,37,56,57]. Notably, *Lachnospiraceae* and *Lactobacillus* species have been described as radioprotective commensals in the gut [35,37,58,59] and contribute to epithelial barrier maintenance through short-chain fatty acid (SCFA) production and competitive exclusion of pathogens [60–63]. These taxa have also been associated with enhanced tissue repair, including recovery following radiation dermatitis [57,64,65]. In our previous work using the same colonized coHSE model, radiation exposure induced epidermal remodeling while preserving overall tissue architecture, suggesting an adaptive response of the epidermis. Together, these observations are consistent with the possibility that microbial shifts reflect selection-driven ecological adaptation favoring taxa supporting tissue protection and recovery, although causal mechanisms remain to be determined.

Consistent with these microbial changes, corresponding metabolic alterations were detected in pathways associated with SCFA metabolism, natural moisturizing factor (NMF)–related amino acid pathways, energy metabolism, oxidative stress responses, and inflammatory lipid signaling across both models. These signatures align with previous metabolomic studies of irradiated mouse and human skin [57,66,67] as well as clinical metabolomics analyses linking metabolites levels such as alanine, aspartate, inosine, and thymine, along with perturbations in pentose and glucuronate interconversions and glutathione metabolism, to the severity of radiotherapy-induced skin reactions [68], several of which were also identified among the radiation-responsive metabolites detected in the present study.

The stratum corneum barrier depends on a lipid matrix composed of ceramides, cholesterol, and free fatty acids [69], and disruption of this permeability barrier triggers compensatory lipid remodeling, including increased fatty acid synthesis by keratinocytes to restore epidermal homeostasis [70,71] and protect against radiation-induced skin injury [72]. In swab samples, unsaturated fatty acids predominated in controls whereas saturated fatty acids were enriched at 1 Gy. However, interpretation of lipid saturation patterns remains complex, as decreased monounsaturated fatty acid species such as C16:1 and C18:1 have been associated with impaired epidermal barrier integrity and increased susceptibility to *Staphylococcus aureus* infection in human skin [73].

Consistently enriched in both models, DAG and TAG lipid classes play central roles in epidermal barrier biology and cellular stress responses. Mechanistically, radiation-induced ROS can activate phospholipase C, promoting DAG accumulation. DAG supports epidermal barrier function and TAG synthesis via DGAT enzymes, processes that are critical for epidermal differentiation [74,75] and have also been implicated in radiation resistance in tumor cells [76]. Hydrolysis of TAGs generates FFAs required for ω-O-acylceramide synthesis, a key step in formation of the cornified lipid envelope of corneocytes. Disruptions in TAG/DAG balance and DGAT activity are associated with severe barrier abnormalities, and dysfunction of enzymes regulating TAG synthesis and degradation can trigger inflammatory skin disorders in humans and mice [77–79]

Beyond their structural role, DAGs also function as signaling lipids, activating protein kinase C (PKC) and influencing inflammatory signaling pathways [80]. Phosphorylation of DAG by diacylglycerol kinase (DGK) generates phosphatidic acid (PA), which was enriched at 4 Gy and is associated with proliferative and survival pathways including mTOR and Hippo signaling, as well as conversion to lysophosphatidic acid (LPA), a pro-fibrotic mediator linked to cell survival and radioresistance [81,82]. PA also serves as a key precursor for glycerophospholipids including PC, PE, and PS [80,83]. Consistent with this network-level remodeling, perturbations in PC/PE metabolism and enrichment of cardiolipin species, which have been associated with mitochondrial stress responses and mitophagy [84–87], further support the presence of coordinated metabolic and structural adaptations following radiation exposure.

### Molecular signatures and translational potential

Multi-omic correlations in the mice revealed molecular and microbial features enabling radiation exposure group separation (AUC > 0.8) and identified a reproducible panel of radio-responsive metabolites across cohorts. Two candidate metabolite panels were identified for future skin-based biodosimetry investigation: one panel of conserved radiation-responsive metabolites distinguishing exposed from non-exposed mice (Fig 7), and a second panel of dose-responsive metabolites discriminating 1 Gy from 4 Gy exposure (Fig 8). These panels included oxidative stress markers (e.g., 8-hydroxyadenine, 8-hydroxy-2′-deoxyguanosine, 20-hydroxy-PGF2α), NMF-related metabolites (e.g., lactic acid, L-serine, L-histidine, D-aspartic acid), and metabolites linked to cardiovascular injury (e.g., 3-nitrotyrosine, glutamine, geranyl-PP). Similitudes in perturbation patterns across cohorts, provide additional support for the reproducibility of the radiation-associated metabolic signatures in the skin. Collectively, these findings suggest that skin molecular changes reflect both tissue stress and radiation dose, supporting the potential of skin swab profiling as a biodosimetry approach. Future studies including a wider range of radiation doses and exposure scenarios will be important to further characterize dose–response relationships and improve the generalizability of skin-based biodosimetry signatures.

Several known radiation-responsive metabolites, including citric acid, creatine, taurine, xanthine, hypoxanthine, and uric acid, were consistently altered in skin swab samples after irradiation [88]. Many of these molecules are linked to oxidative stress pathways; for example, uric acid is generated by xanthine oxidoreductase, which metabolizes hypoxanthine and xanthine during oxidative stress responses [89,90]. In addition, several discriminating metabolites corresponded to NMF components derived from filaggrin degradation, including arginine, serine, proline, glutamic acid, and urocanic acid, which play critical roles in epidermal hydration and barrier function [23,48,91–93]. Dose-dependent increases in these metabolites suggest that skin barrier molecular components may provide quantitative biodosimetric signals, consistent with guidelines linking radiation dose to cutaneous injury severity.

In addition to biodosimetry applications, an appealing concept lies in monitoring internal health status through skin molecular cues. Growing evidence supports the existence of a gut–skin axis, a bidirectional pathway by which intestinal microbiota influence skin and systemic inflammation via immune and metabolic signaling [40,94,95]. Among key microbial families, *Lachnospiraceae* stand out for producing SCFAs that reinforce epithelial barrier integrity and reduce cardiovascular risk [96–98]. These findings raise the possibility that skin responses to radiation-induced stress may mirror internal physiological perturbations, including cardiovascular injury, shaped in part by the gut microbiota. Of note, cutaneous manifestations typical of radiation exposure, such as edema, telangiectasia, and ulceration, also commonly appear in various cardiovascular diseases, particularly those with inflammatory components [99–103]. The skin, involved in thermoregulation, vascular tone, and immunity, receives ~10% of cardiac output, making it biologically relevant for reflecting systemic injury signals. Interestingly, s Several discriminating metabolites identified here (e.g., phenylalanine, 3-nitrotyrosine, hypoxanthine, succinate, and trimethyllysine) have also been implicated in cardiovascular dysfunction [104–106]. Others, such as acylcarnitines, inosine, xanthine, and taurine, correlate with myocardial ischemia, remodeling, or heart failure progression [104,^105^,^107^–]. Interestingly, skin levels of metabolic markers such as cholesterol and carotenoids have been associated with cardiovascular disease risk [106,108]. These observations suggest that skin alterations reflect broader systemic conditions and might serve as a non-invasive window to assess internal damage. Supporting this concept, optical skin readouts have been shown to predict cardiovascular disease and mortality risk in both murine models and human cohorts [109–111]. Further studies, including targeted imaging and histopathology, are needed to confirm associations between omic profiles and radiation-induced injury.

### Integration and robustness

Radiation exposure induces coordinated molecular responses across multiple biological layers, including host metabolism, lipid remodeling, and microbial community dynamics. While each omic layer independently captures aspects of radiation response, these signals may exhibit intrinsic variability due to biological heterogeneity, temporal dynamics, and environmental influences. Multi-omic integration provides a framework to identify coherent, cross-domain molecular signatures that reflect systemic biological responses rather than isolated fluctuations within individual datasets. By leveraging covariance structures between metabolomic, lipidomic, and metagenomic profiles, integrative approaches such as DIABLO enable identification of biomarker panels that remain robust despite variability within any single omic layer.

Importantly, both the skin microbiome and metabolome are inherently variable across individuals and time, reflecting dynamic host–microbe interactions and physiological regulation. Rather than representing a limitation, this variability underscores the importance of integrative analysis, which can identify consistent biological response patterns that emerge across molecular domains. By focusing on coordinated multi-omic signatures rather than single features, this approach enhances robustness and improves the reliability of detecting radiation-associated biological effects, particularly in translational biodosimetry contexts where inter-individual variability is expected.

### Limitations

Several limitations should be acknowledged. Although an independent validation cohort was included, the discovery-phase sample size remained modest, which may affect feature stability. While key metabolite perturbations were reproducible across cohorts, classification performance remained moderate, highlighting the distinction between reproducible biological signals and generalizable predictive models.

Although microbiome inoculation incorporated multiple human donors, some demographic imbalance was present, and the study design cannot fully disentangle direct radiation effects from secondary ecological or housing-related influences. Accordingly, microbial shifts n taxa such as *Lachnospiraceae* and *Lactobacillales* should be interpreted as associations, and the 16S approach provides limited functional resolution, requiring future validation using metagenomics and targeted metabolite measurements.

Supervised integration methods such as DIABLO and sPLS-DA may overestimate performance when applied within the same dataset used for feature selection. To mitigate this, we applied sparsity constraints, cross-validation, permutation testing, and independent validation; however, larger cohorts and alternative modeling strategies will be needed to confirm robustness. In addition, pooling early time points (days 1–7) enabled identification of dose-associated signatures relevant to operational settings but does not resolve temporal dynamics, which should be addressed in future longitudinal studies.

To translate these findings into practical human biodosimetry tools, several steps are required: (1) validation in human cohorts with known exposures; (2) evaluation of key confounders; (3) model calibration and temporal stability assessment; (4) development of targeted assays; and (5) establishment of standardized and scalable workflows. Emerging transdermal sensing technologies, including Raman spectroscopy and wearable microneedle-based sensors, provide a promising path toward longitudinal and potentially real-time monitoring of radiation-responsive biomarkers [112,113]. Together, these advances support future integration of validated radiation-responsive biomarkers into wearable biodosimetry platforms that could complement existing cytogenetic and blood-based approaches.

In conclusion, this study demonstrates for the first time that multi-omic profiling of skin swabs can be used to monitor radiation exposure and has identified two metabolite panels with potential for future skin-based biodosimetry. Although skin metabolomics in humans is currently constrained by the need for laboratory-based processing, emerging optical technologies, such as Raman or infrared spectroscopy, already proven for real-time, non-invasive detection of skin biomarkers [114–120] can enable biodosimetry [121,122] in configurations compatible with various clinical settings, emergency response, or spaceflight, and should be further explored in the future, anchored by multi-omic studies such as this one to support translational application.

## Supporting information

S1 Fig Pathway enrichment analysis of metabolites differentially altered between 1 Gy and 4 Gy exposed coHSE samples (ORA, KEGG library), highlighting metabolic pathways contributing to radiation dose discrimination. (TIF)

S2 Fig Spectral abundance profiles of radio-responsive metabolites in coHSE samples, supporting identification of conserved metabolic signatures associated with radiation exposure. (TIF)

S3 Fig Spectral abundance of natural moisturizing factor (NMF) components in coHSE following radiation exposure, illustrating alterations in metabolites associated with epidermal barrier homeostasis. (TIF)

S4 Fig Lipid reaction network analysis showing suppressed and activated lipid transformations in coHSE following radiation exposure, highlighting remodeling of lipid metabolic pathways. (TIF)

S5 Fig Diagnostic plots of DIABLO multiblock sPLS-DA model performance for the second latent component (component 2) in mouse skin following radiation exposure. (TIF)

S6 Fig Lipid reaction network analysis showing suppressed and activated lipid transformations in mouse skin following radiation exposure. (TIF)

S7 Fig Receiver operating characteristic (ROC) curve analysis evaluating the performance of NMF metabolites and candidate biomarker panels for discriminating non-exposed (0 Gy) versus exposed (1 Gy and 4 Gy) samples, and for distinguishing 1 Gy from 4 Gy exposure. (TIF)

S1 Tables A) Human donor microbiome diversity used for coHSE inoculation, showing the distribution of samples contributed by each donor across radiation exposure groups; B) Demographic distribution of microbiome donors (sex and age ranges) contributing to the coHSE inoculation. (TIF)

S2 Table MS/MS fragment matches used for metabolite identification in mouse skin swab samples. Metabolites were annotated based on matches to pure standards (Metabolomics Standards Initiative Level 1) or spectral libraries (HMDB and METLIN; Level 2). Unmatched fragments are indicated by “//”.
(CSV)

S3 Table Differentially abundant microbial taxa (Cluster 1) identified in mouse skin following 1 Gy and 4 Gy radiation exposure. (XLSX)
