# Supplementary material for: Monitoring radiation exposure through skin swab multi-omic profiling
Source: PLoS One. 2026 Aug 5;21(8):e0354734. doi: 10.1371/journal.pone.0354734 (PMC13440882; doi:10.1371/journal.pone.0354734)
Supplement: Supplementary file 1 [file pone.0354734.s013.docx]

Dear Editor and Reviewers,

We sincerely thank you for your careful evaluation of our manuscript and for the constructive and insightful comments. We have carefully revised the manuscript to address all concerns raised. These revisions have strengthened the clarity, rigor, and translational framing of the study.

Below, we provide a detailed, point-by-point response to each reviewer’s comment. All changes have been incorporated into the revised manuscript and are indicated accordingly.

**Review Comments to the Author**

**Reviewer #1:**

Summary
This manuscript presents a multi-omic study proposing skin swab–based metabolomic, lipidomic, and microbiome profiling as a non-invasive biodosimetry platform for ionizing radiation exposure. Using colonized human skin equivalents and mouse models exposed to 0, 1, or 4 Gy of x-rays, the authors apply DIABLO-based multiblock sPLS-DA to identify correlated, dose-responsive molecular and microbial signatures. While the experimental execution is extensive and the datasets are large, the study ultimately prioritizes multivariate discrimination over biological rigor. Many conclusions rely heavily on supervised classification, post-hoc feature selection, and pathway over-interpretation, without sufficient control for overfitting, temporal dependence, or biological confounding. As a result, the manuscript reads more as a methodological showcase than a validated biodosimetry framework, and its translational claims are substantially overstated.

Strengths
The study addresses a relevant and timely problem in radiation biology: the need for rapid, non-invasive biodosimetry. The use of skin swabs is innovative and practically attractive, and the parallel analysis of human skin equivalents and mouse skin is conceptually sound. The experimental workflows for metabolomics, lipidomics, and 16S profiling are technically competent and well described. The authors make effective use of modern multi-omic integration tools and present a large amount of data in a transparent manner. The inclusion of ROC analyses and dose-resolved comparisons demonstrates an effort to evaluate diagnostic performance rather than relying solely on descriptive trends.

Recommendations
-The central weakness of the manuscript lies in its analytical strategy. The study relies almost exclusively on supervised sPLS-DA (DIABLO) to define “radiation-responsive” features, yet provides insufficient safeguards against overfitting.

Response: We thank the reviewer for raising this important point. In the revised manuscript, we clarify that DIABLO-based sPLS-DA was used strictly as an exploratory framework to identify coordinated, dose-responsive features within the discovery cohort, rather than to build a predictive model. Accordingly, AUC values derived from DIABLO are now explicitly described as reflecting within-cohort separation and not out-of-sample predictive performance.

To address concerns regarding overfitting and generalizability, we implemented an independent validation analysis using a separate mouse cohort not used for feature selection or model fitting. In this cohort, the candidate metabolite panel reproduced consistent metabolic perturbations, with moderate classification performance (AUC ≈ 0.70–0.72) but strong concordance in effect size (R = 0.87), indicating that the identified features capture reproducible biological responses across datasets. These results, now presented in Fig. 9, shift the emphasis of the study from predictive performance toward reproducibility and biological consistency:

“Fig 9. Independent validation of radiation-responsive skin metabolite panels and dose performance evaluation. A) Heatmap showing relative abundance of candidate metabolites across the validation cohort for 0 Gy, 1 Gy, and 4 Gy exposure groups, demonstrating conserved dose-dependent metabolic patterns. B) Receiver operating characteristic (ROC) analyses evaluating classification performance of the metabolite panel for distinguishing non-exposed from exposed samples and for dose discrimination, with permutation testing to assess statistical significance. C) Replication analysis comparing metabolite fold changes between discovery and validation datasets. Absolute fold-change effects were strongly correlated (R = 0.87), indicating reproducible magnitude of metabolic alterations across cohorts.”

-Sample sizes per group are small relative to the number of features, time points are pooled rather than modeled explicitly, and the same datasets are repeatedly used for feature selection and performance evaluation.

Response: We thank the reviewer for this comment. To address concerns related to sample size, feature selection, and potential overfitting, we implemented an independent validation strategy using a separate mouse cohort not used during feature selection or model fitting. As shown in Fig. 9, the identified metabolite panel reproduced consistent dose-dependent perturbations, with moderate classification performance but strong concordance in effect sizes between cohorts (R = 0.87), supporting the robustness of the underlying biological signal.

We have also clarified in the Methods the rationale for pooling early post-irradiation time points (days 1–7), which was performed to identify dose-dependent signatures conserved across the early response window relevant to operational biodosimetry scenarios, rather than to model temporal dynamics. This limitation is now explicitly acknowledged, and longitudinal modeling is discussed as an important direction for future work.

-High AUC values reported for refined metabolite panels, particularly those approaching 0.99, are therefore not convincing without independent validation, permutation testing, or nested cross-validation. At minimum, the authors should demonstrate model robustness through repeated resampling and clearly separate training and evaluation steps.

Response: We agree with the reviewer that AUC values derived from supervised analyses can overestimate generalizable performance when feature selection is performed on the same dataset. ROC curves and AUC values were estimated using stratified 5-fold cross-validation, with all preprocessing and feature selection performed within each training fold to prevent information leakage. Model performance was additionally evaluated using permutation testing (n=100 label shuffles) to assess whether cross-validated discrimination exceeded chance expectations.

→Updated figures caption with cross-validated AUC + permutation test results:

Lipidomics (Figure 5 and 6)

- 4 Gy vs 0 Gy: AUC 95% CI 0.490–1.000, 100 permutation p = 0.158
- 1 Gy vs 0 Gy: AUC 95% CI 0.302–1.000, 100 permutation p = 0.248

Metagenomic (Figure 5 and 6)

- 4 Gy vs 0 Gy: AUC 95% CI 0.142–1.000, 100 permutation p = 0.337
- 1 Gy vs 0 Gy: AUC 95% CI 0.167–1.000, 100 permutation p = 0.356

Importantly, in the revised manuscript, we have clarified that AUC values reported for DIABLO-derived features reflect within-cohort separation rather than out-of-sample predictive accuracy. To more rigorously assess robustness, we prioritized independent validation in a separate mouse cohort (Fig. 9). While classification performance was moderate in this setting, the strong concordance in metabolite effect sizes between cohorts (R = 0.87) supports the reproducibility of the identified signatures.We have accordingly tempered claims related to classification performance and reframed the analysis to emphasize reproducibility rather than predictive optimization.

-Temporal structure is inadequately handled. Samples collected at 1, 3, and 7 days post-irradiation are pooled for many analyses, despite the well-known time dependence of radiation responses. Pooling obscures whether identified signatures represent true dose effects or simply reflect differences in recovery kinetics. A proper longitudinal analysis, or at least stratified presentation by time point, is necessary to support claims of dose-specific signatures.

Response: We acknowledge that radiation responses can be time dependent. We agree that a dedicated longitudinal modeling framework would provide additional mechanistic insight and note this as an important future extension. In this study, samples collected at days 1, 3, and 7 were pooled for primary discrimination analyses to identify conserved early biodosimetric signatures within the first week post-exposure, consistent with emergency response timeframes. To clarify this rationale, we have:

- Explicitly stated this design choice in the Methods section.
- Added supplementary clarification that time-stratified exploratory analyses yielded consistent dose-directional trends (now referenced in the revised manuscript).

“Because radiation responses exhibit temporal dynamics, samples collected at days 1, 3, and 7 post-irradiation were pooled for primary dose discrimination analyses to identify molecular and microbial signatures that are conserved across early post-exposure time points and primarily reflect radiation dose rather than time-dependent biological recovery processes. This time window reflects operational biodosimetry scenarios where rapid exposure classification is required regardless of the exact time since exposure within the early response phase. By reducing the influence of temporal variability, this strategy enables identification of biodosimetric features capable of discriminating exposure level independently of post-exposure temporal progression during the first week following irradiation. Dedicated longitudinal modeling to resolve temporal trajectories and fully separate dose-specific from time-dependent effects represents an important direction for future investigation.”

-Biological interpretation consistently overreaches the data. Pathway enrichment results are derived from putatively identified metabolites selected via supervised models, yet are discussed as evidence of coordinated biological programs (e.g., barrier disruption, mitochondrial dysfunction, systemic cardiovascular risk). These interpretations are speculative in the absence of orthogonal validation such as histology, barrier function assays, inflammatory readouts, or targeted biochemical measurements. The frequent extension of skin molecular changes to systemic health implications is particularly problematic given that no systemic endpoints were measured.

**Response:** We thank the reviewer for this important comment and agree that pathway enrichment results should be interpreted cautiously. Accordingly, we have moderated causal language throughout the Discussion and clarified that pathway-level interpretations represent biologically consistent hypotheses rather than direct functional validation. We also note that most metabolites included in the pathway analysis were confidently annotated at Metabolomics Standards Initiative (MSI) Level 1 or Level 2 using authentic standards or spectral library matches (HMDB/METLIN), which increases confidence in metabolite identification while still warranting cautious biological interpretation (S2 table).

To provide additional biological context, we now reference orthogonal evidence from our previous work using the same colonized human skin equivalent (coHSE) model, where histological analyses demonstrated radiation-induced epidermal remodeling while preserving overall tissue architecture. We also note that independent clinical metabolomics studies have reported alterations in similar metabolic pathways (e.g., alanine–aspartate–glutamate metabolism, pentose and glucuronate interconversions, glutathione metabolism) associated with the severity of radiotherapy-induced skin reactions in breast cancer patients.

Together, we believe these observations support a repair-driven epidermal response to radiation exposure, while highlighting the need for future functional validation through targeted biochemical, histological, and barrier function studies.

-Microbiome analyses raise similar concerns. Changes in taxa such as Lachnospiraceae and Lactobacillales are repeatedly framed as radioprotective or reparative, but the study design cannot distinguish direct radiation effects from secondary ecological shifts or housing effects. Moreover, 16S-based taxonomic resolution is insufficient to support functional claims without complementary metagenomic or metabolomic corroboration.

Response: We thank the reviewer for this comment. In the revised Discussion, we now acknowledge that the study design does not allow us to distinguish direct radiation effects from secondary ecological or housing-related influences on the microbiome. We also clarify that the 16S rRNA sequencing approach provides limited functional resolution and note that future studies integrating metagenomics and metabolomics will be needed to validate the functional significance of these microbial shifts.

Added paragraph in the discussion: “Interpretation of microbiome changes should also be approached with caution. Because the study design cannot fully disentangle direct radiation effects from secondary ecological shifts or housing-related influences, observed alterations in taxa such as Lachnospiraceae and Lactobacillales should be interpreted as associations rather than evidence of radioprotective or reparative functions. Furthermore, the 16S rRNA sequencing approach used here provides limited functional resolution, and future studies integrating shotgun metagenomics, metabolomics, or targeted microbial metabolite measurements will be required to clarify the mechanistic contribution of microbiome changes to radiation responses.”

-While the integration of multi-omic skin swab data is technically impressive, the study does not yet establish a deployable biodosimetry tool. Claims regarding real-time monitoring, emergency response utility, or spaceflight applications are premature and should be clearly labeled as future directions rather than inferred outcomes.

Response: We agree with the reviewer that the present study does not yet establish a deployable biodosimetry platform. The manuscript has been revised to clarify that the current work represents a proof-of-concept demonstration of radiation-associated molecular signatures detectable from skin swabs. Statements regarding real-time monitoring, emergency response applications, or spaceflight relevance have been reframed as future directions within a translational roadmap section outlining the steps required for clinical and operational implementation.

Conclusion
This is a data-rich and technically sophisticated study, but it is analytically and interpretively overextended. The reliance on supervised multivariate methods without adequate validation, the pooling of temporally distinct samples, and the speculative biological extrapolations substantially weaken the manuscript’s conclusions. As currently presented, the work does not convincingly demonstrate a robust, generalizable skin-based biodosimetry platform. Major restructuring of the analysis, substantial tempering of claims, and additional validation would be required to support publication.

Response: We thank the reviewer for this thoughtful and constructive assessment. In response, we have substantially revised the manuscript to better align the analytical strategy, interpretation, and scope of conclusions with the data.

Specifically, we clarified the exploratory role of supervised multi-omic integration, explicitly distinguished between discovery and validation phases, and added an independent validation cohort demonstrating reproducible metabolite perturbations across datasets (Fig. 9). We also tempered claims related to predictive performance and translational applicability and restructured the Discussion to clearly distinguish observation from interpretation while acknowledging key limitations, including pooled time points and lack of orthogonal functional validation. Collectively, we believe that these revisions reposition the study as a rigorous, data-driven proof-of-concept demonstrating reproducible skin-based molecular responses to radiation exposure, while outlining the necessary steps for future validation and translation.

**Reviewer #2:**

This study systematically investigates the dose-dependent response of skin to ionizing radiation by integrating multi-omics (microbiome, metabolome, lipidome) and cross-model (human skin equivalent coHSE, mouse) approaches. The research design is novel, and the data volume is substantial. It is the first to propose a "host-microbe co-metabolism" framework for radiation response at the skin level and to screen biomarker combinations with high diagnostic potential, which holds significant value for the development of radiation biodosimetry. However, the paper has deficiencies in the rigor of experimental details, the depth of data interpretation, and logical coherence, requiring substantial revisions to support its important conclusions.

Response: We thank the reviewer for this thoughtful assessment. In response to these concerns, we substantially revised the manuscript to temper interpretative claims and clarify the scope of our conclusions. We also added additional safeguards against overfitting, including independent validation using a separate mouse cohort and replication analysis demonstrating strong concordance in metabolite perturbation magnitudes between discovery and validation datasets (R = 0.87; new Fig 9). The Discussion has been restructured to better distinguish observations from interpretation, to explicitly acknowledge analytical limitations (including pooled time points), and to frame the study as a proof-of-concept for skin-based biodosimetry rather than a finalized platform. We believe these revisions substantially strengthen the analytical rigor and balance of the manuscript.

1.P150: Briefly explain why the control group has a larger sample size.

Response: We thank the reviewer for this comment and would like to clarify the sample sizes used in each experimental model. In the coHSE model, group sizes were 0 Gy (sham, n = 8), 1 Gy (n = 6), and 4 Gy (n = 6). The slightly larger sham group was intentionally included to improve characterization of baseline variability, which was expected to be potentially greater than in mice due to the intrinsic diversity of human-derived microbial communities. Because the control group serves as the primary reference for multiple dose comparisons and normalization steps, increasing its size enhances statistical stability without introducing bias, providing a more robust reference condition for downstream comparative and integrative multi-omic analyses.

In contrast, in the mouse study, animals were randomly assigned to dose groups with equal sample sizes and balanced sex distribution: 0 Gy (sham, n = 6), 1 Gy (n = 6), and 4 Gy (n = 6). Equal group sizes were maintained in accordance with standard experimental design principles and to adhere to ethical guidelines for animal research, specifically the principle of reduction, which aims to minimize the number of animals used while ensuring adequate statistical power. Therefore, no imbalance in group size exists in the mouse experiments.

Updated Material and Methods section: “A slightly larger sham group was included in the coHSE model to improve characterization of baseline variability inherent to human-derived microbial communities and to provide a robust reference for downstream comparative and integrative analyses.”

2.Briefly state the rationale for selecting doses of 1 Gy and 4 Gy.

Response: The 1 Gy and 4 Gy doses were selected to represent exposure levels relevant to both experimental radiation biology and real-world exposure scenarios in the coHSE and mouse skin models. A dose of approximately 1 Gy falls within the range of individual fraction doses commonly used in radiotherapy (typically 1.8–2 Gy per fraction) and overlaps with exposure levels that may occur in occupational, accidental, or spaceflight contexts. A dose of 4 Gy corresponds to the cumulative effect of multiple radiotherapy fractions or localized acute exposures and extends into exposure levels associated with clinically biological effects, as recognized in radiation emergency response and biodosimetry frameworks, including IAEA and BARDA guidelines. For clarity, we added this rational for dose selection in the introduction:

“Radiation doses of 1 Gy and 4 Gy were selected to reflect exposure levels relevant to fractionated radiotherapy and radiation emergency scenarios, spanning a biologically and clinically relevant range recognized in biodosimetry frameworks. This dose interval enables evaluation of dose-dependent molecular responses and assessment of the capacity of skin-based multi-omic profiling to detect and discriminate radiation exposure under realistic and translationally relevant photon exposure conditions.”

3.Shaving itself is a strong physical and mildly traumatic stimulus that can significantly disrupt the skin barrier, alter the local microenvironment (e.g., temperature, humidity), and trigger skin inflammation and repair responses. This greatly perturbs the structure of the skin microbial community before sampling, making it unrepresentative of the "steady-state" skin flora, instead reflecting a "post-perturbation recovery" state. The animal experiment design of "shaving 24 hours before sampling" severely interferes with skin microbial homeostasis. This constitutes a fundamental flaw that must be modified or provide strong reasons.

Response: We thank the reviewer for raising this important point regarding the potential impact of shaving on skin barrier integrity and microbiome composition. We agree that shaving can transiently perturb the skin barrier and local microenvironment. To address this potential source of variability, the timing of shaving was prospectively optimized as part of our original experimental design and incorporated into a standardized operational procedure applied equally across all animals. Specifically:

• All animals were shaved at least 24 hours prior to irradiation to allow initial recovery of the skin surface.
• Skin swab samples were collected at 1, 3, and 7 days post-irradiation, ensuring a minimum of 48 hours between shaving and the first post-irradiation sampling.
• All experimental groups, including sham and irradiated animals, underwent identical preparation and handling procedures.

Clarification has now been added to the Methods section:

“Skin swabs were collected from a 1.5 × 6 cm area on the dorsal skin, which was shaved at least 24 hours prior to irradiation to allow initial microbial recovery, with a minimum of 48 hours between shaving and the first post-irradiation sample collection at 1, 3, and 7 days. The timing of shaving was prospectively optimized during experimental planning and incorporated into a standardized operational procedure to minimize transient perturbations of the skin barrier and microbiome while ensuring consistent and reproducible sampling conditions across all animals.”

4.The temporal design alignment between the human skin equivalent (coHSE) and mouse experiments is excellent, and the use of the same irradiation device is a major advantage. However, the inconsistency in key irradiation physical parameters (energy, filter, dose rate) means the radiation received in the two experiments differs in physical characteristics. The rationale for selecting these parameters should be briefly explained.

Response: We thank the reviewer for highlighting this important point. The differences in beam energy, filtration, and dose rate reflect the use of different irradiation equipment across two facilities, each operating under its standard calibrated configuration optimized for the respective biological model and experimental setup.

Both irradiators operated at 300 kVp and 320 kVp, respectively, which fall within well-established and appropriate energy ranges for small animal irradiation, skin studies, and in vitro radiation biology. These closely matched beam energies ensure comparable photon exposure conditions across the coHSE and mouse models.

While beam conditioning parameters such as filtration influence beam energy spectrum, the use of a softer orthovoltage beam in mice would result in a small enhancement to bone marrow dose (see fig 3 of [<https://pubmed.ncbi.nlm.nih.gov/32315248/>]) and to a somewhat steeper depth-dose curve. As we are only measuring effects in skin, neither of these are expected to affect our results.

In terms of dose rate, dose rate effects are normally seen when varying dose rates by orders of magnitude (e.g. at low dose rates on the order of Gy/day, repair of sublethal damage plays an important part, whereas at dose rates of 10s of Gy/sec the FLASH effect becomes important). In this study the difference in dose rate is small (0.8 Gy/min vs 1.3 Gy/min) and a biological difference is not expected.

Importantly, in both experimental systems, the delivered dose was precisely measured and verified using NIST-traceable ionization chambers, ensuring accurate and reproducible dosimetry. This clarification has now been added to the Methods section:

- coHSE procedures: “This irradiation system operates within well established and appropriate X-ray range for in vitro radiation biology and skin irradiation studies. Delivered dose was verified using NIST-traceable dosimetry, ensuring accurate and reproducible absorbed dose (Gy), the biologically relevant parameter reflecting energy deposition in tissue.”
- Mouse procedures: “This system also operates within appropriate voltage for small animal irradiation and skin radiation studies. Differences in filtration and dose rate reflect facility-specific equipment configurations. However, in both experimental systems, delivered dose was verified using NIST-traceable dosimetry, ensuring accurate and reproducible absorbed dose (Gy), thereby enabling valid comparison of dose-dependent biological responses across models.”

5.The current statistical description is the weakest part of the methods section. It fails to match the complex time-dose multi-factorial design of your study. It is recommended to rewrite the statistics section with a more rigorous and detailed description.

Response: We thank the reviewer for this important comment. The statistical analysis section has been substantially rewritten to more accurately reflect the multi-factorial design of the study and to improve methodological rigor and transparency.

Specifically, we now (i) explicitly justify the pooling of early post-irradiation time points (days 1–7) to focus on dose-dependent signatures relevant to operational biodosimetry, (ii) provide a detailed description of data preprocessing, including global signal normalization, QC-based LOESS drift correction, and ComBat batch correction, (iii) clarify the statistical tests used for univariate analyses, (iv) explicitly distinguish between the exploratory discovery phase (DIABLO-based multi-omic integration) and the independent validation phase, and (v) detail the validation strategy, including fold-change concordance analysis and ROC/permutation testing in a separate cohort not used for feature selection or model fitting. Importantly, we now clarify that AUC values derived from DIABLO reflect within-cohort separation rather than out-of-sample predictive performance, and that generalizability was specifically assessed using an independent validation cohort. Together, these revisions provide a more rigorous and transparent description of the analytical framework and better align the statistical methods with the study design and objectives.

6.P445-447: It is suggested to change to: "1 Gy radiation exposure was primarily associated with the activation of carbohydrate metabolism pathways, while concurrent changes in the abundance of microbial taxa such as *Lachnospiraceae* were observed." Lines 565-568

→ We corrected as suggested.

7.P474: Confirm the accuracy of the lipid nomenclature for "TAG(52:7)-FA(16:0), and (-2)Cl66:3-FA(16:1)".

Response: TAG(52:7)-FA(16:0) represents a triacylglycerol species identified based on precursor mass and confirmed by MS/MS fragment corresponding to FA(16:0). This notation reflects fragment-supported lipid identification and is consistent with standard lipidomics annotation practices. The abbreviation “CL” refers to cardiolipin species, and lipid annotations in the figures reflect instrument-derived feature identifiers used during targeted lipidomics analysis with specific multiple reaction monitoring transitions. To improve clarity while preserving consistency with the analytical output, we have added explicit definitions of cardiolipin nomenclature and instrument-specific annotations in the figure legends and Methods section. These clarifications ensure alignment with standard lipid class conventions while maintaining traceability to the original lipidomics dataset.

- Figure legends: “CL denotes cardiolipin species, reported as total carbon number and double bonds (e.g., CL66:3). Instrument-specific annotations (e.g., “(-2)”) correspond to feature identifiers used in lipidomics analysis.”
- Methods section: “Lipid species were annotated using standard lipid class abbreviations (e.g., TAG, CL). Instrument-specific identifiers and charge annotations (e.g., “(-2)”) were retained in figures for consistency with lipidomics software outputs and are defined in figure legends.”

8.P500: Change "1Gy" to "4Gy".

→ We corrected the typo

9.Much of the Discussion section reiterates specific observations already detailed in the Results, without sufficient interpretation, synthesis, or attribution of new meaning. The Discussion should avoid being a secondary narration of the results. It is recommended to significantly condense the restatement of specific results and instead emphasize the new concepts, models, or insights distilled from these results.

Response: We thank the reviewer for this helpful comment. We agree that the Discussion should focus on interpretation and conceptual synthesis rather than restating detailed observations from the Results. Accordingly, we substantially revised and condensed the Discussion section to reduce repetition of specific quantitative findings and instead emphasize the broader biological interpretation and conceptual implications of the results. In the revised manuscript, we reorganized the Discussion into thematic sections highlighting the biological significance of microbiome shifts, lipid remodeling, and metabolite alterations, as well as their implications for skin barrier biology, host–microbiome interactions, and potential biodosimetry applications. We also strengthened the synthesis across experimental models (coHSE and mouse skin) to emphasize conserved multi-omic response patterns rather than reiterating individual result descriptions.

**Reviewer #3:**

Overall comment:
This study demonstrated that radiation caused dose-dependent shifts in the skin microbiome in both human skin equivalents and mice. Several taxa - especially Lachnospiraceae/Oscillospiraceae/Bacillaceae - emerged as candidate biomarkers for non-invasive radiation biodosimetry. Although the study demonstrates the potential of skin swab multi-omic profiling for biodosimetry, the translational application of these findings to human settings remains uncertain. Additional research is needed to validate the findings in human subjects and develop practical tools for real-time monitoring. The study uses relatively small sample sizes for both coHSEs and mouse models, which may limit the statistical power and generalizability of the results. Additionally, the study pools microbial samples from human donors to inoculate coHSEs, which may not fully replicate the diversity and complexity of individual human skin microbiomes.

Specific comments

Abstract

- Please provide more details on sample sizes of human skin equivalents and murine models and how the findings can be applied to human biodosimetry.

**Response:** We have revised the Abstract to include explicit sample sizes for both models (coHSE and mice) and to better frame translational relevance while remaining appropriately cautious. We now state that these findings support feasibility of non-invasive skin swab profiling as a candidate biodosimetry approach and that translation requires human cohort validation:

“Using colonized human skin equivalents (coHSE; 0 Gy n=8, 1 Gy n=6, 4 Gy n=6) and mice (n=6/group) models […] These data support the feasibility of skin swab signatures for non-invasive exposure classification, providing a molecular and microbial framework for skin based monitoring measure development and motivating validation in human cohorts for real-world.”

Introduction

- Please provide more detailed comparisons of existing biodosimetry methods and their shortcomings.

Response: The Introduction already summarizes the principal biodosimetry approaches currently in use, including cytogenetic methods (dicentric chromosome assay, CBMN), molecular biomarkers (γ-H2AX, gene expression), physiological indicators (CBC), and physical dosimetry techniques (EPR, OSL), and highlights their key limitations, including labor intensity, delayed turnaround time, invasiveness, limited scalability, and lack of real-time biological response assessment. (Lines 72-87). These limitations are particularly relevant in emergency triage and spaceflight contexts, where rapid, non-invasive, and scalable biodosimetry tools are critically needed.

To further clarify these distinctions without overextending the Introduction, we have slightly modified the relevant section to more explicitly emphasize practical deployment constraints, including sample collection requirements, throughput limitations, and limited suitability for longitudinal monitoring. The section describing current biodosimetry approaches now reads as:

“The gold standard approaches include cytogenetic techniques such as dicentric chromosome analysis and the cytokinesis-block micronucleus assay (CBMN). Theses assays, while highly accurate, involve labor-intensive, time-consuming techniques that typically require specialized laboratory infrastructure and several days to generate results, limiting their utility for rapid triage and field deployment during large-scale exposure events or space missions. Furthermore, these approaches also provide limited phenotypic insight that could guide preventive or therapeutic countermeasures. Similarly, alternative blood-based molecular and protein biomarkers methods, such as lymphocyte depletion kinetics via complete blood count (CBC), premature chromosome condensation (PCC), γ-H2AX foci formation, and physical dosimetry using Electron Paramagnetic Resonance (EPR) and Optically Stimulated Luminescence (OSL) of the teeth, require invasive sampling and may not be readily deployable for repeated or large-scale monitoring (6–17), Therefore, there is an unmet need to develop alternative non-invasive biodosimetry approaches rapidly deployable, scalable, and capable of reflecting not only radiation dose but also biological response trajectories.” Lines 72-87

- Please explain why skin swabs are particularly suitable for biodosimetry compared to other non-invasive methods.

Response: We added text emphasizing that skin swabs are (i) minimally invasive, (ii) repeatable over time, (iii) operationally simple, and (iv) capture both host and microbial responses at a tissue interface that is directly exposed and responsive to radiation. We also emphasize practical benefits: low training burden, scalable collection, and compatibility with diverse analytic platforms:

“We recently demonstrated that skin swabs collected from bioengineered human skin equivalents colonized with natural microbial communities and the mouse skin displayed similar taxonomic and metabolomic features in response to radiation exposure (Vitry et al 2025). Operationally simple, skin swabs offer a minimally invasive, low training-burden sampling strategy compatible with diverse analytic platforms that can be repeated frequently, enabling longitudinal assessment. Unlike many biofluid-based approaches, swabs capture responses at a highly accessible and most directly exposed tissue interface and simultaneously measure host metabolites/lipids and microbiome shifts, enabling multi-layered signatures for exposure assessment.”

- Please highlight the need for validation in human subjects and the challenges of translating findings from animal models and coHSEs.

Response: We thank the reviewer for highlighting the importance of translational validation. We have now clarified this point in the Discussion by emphasizing that the present study represents a proof-of-concept in experimental models (mouse skin and colonized human skin equivalents) and that validation in human subjects will be required before clinical or operational biodosimetry applications can be established.

Specifically, we added a short translational roadmap section outlining key steps for future work, including (1) validation in human cohorts with well-characterized radiation exposure (e.g., radiotherapy patients), (2) evaluation of potential confounders affecting skin microbiome and metabolome profiles, and (3) development of simplified targeted biomarker panels and operational workflows for rapid deployment. We also acknowledge in the limitations section that differences between experimental models and human physiology represent an important challenge for translation.


Materials and Methods

- Please provide more details on the diversity of microbial samples used to inoculate coHSEs and how representative they are of human skin microbiomes.

Response: We thank the reviewer for highlighting the importance of donor diversity and representativeness of the microbial inoculum. To enhance the ecological validity and translational relevance of the coHSE model, we used a multi-donor approach incorporating natural inter-individual variability characteristic of human skin microbiomes rather than relying on a single donor-derived microbial community. We have now added detailed information on donor representation and demographics in the revised Methods section and Supplemental Tables:

Summary of microbiome donor representation across coHSE experimental groups:

| **Dose group** | **Number of inoculations** | **Unique donors represented** |
| --- | --- | --- |
| 0 Gy | 8 | 7 |
| 1 Gy | 6 | 5 |
| 4 Gy | 6 | 5 |
| **Total** | **20** | **11** |

Demographic distribution of microbiome donors:

|  | Age range | | |
| --- | --- | --- | --- |
|  | 20- 40 years | 40 - 60 years | Total |
| Females | 1 (9.1%) | 6 (54.5%) | 7 (63.6%) |
| Males | 3 (27.3%) | 1 (9.1%) | 4 (36.4%) |
| Total | 4 (36.4%) | 7 (63.6%) | 11 (100%) |

In total, microbiome inocula used to colonize coHSE models were derived from 11 independent human donors, contributing 20 total inoculations distributed across all experimental dose groups (0 Gy: n = 8; 1 Gy: n = 6; 4 Gy: n = 6). Each dose condition included microbial communities originating from multiple independent donors, and several donors contributed samples across multiple dose groups, thereby reducing potential donor-specific bias.

The donor cohort included both sexes, although females were more represented overall (63.6% female, 36.4% male). Male donors were predominantly represented in the younger adult age group (L=20-40 years), while female donors were mainly dominated by older participant group (U= 40-60 years). This distribution reflects the composition of the available donor pool but still captures inter-individual variability in human skin microbiomes. Importantly, microbiomes from multiple independent donors were included in each experimental group, ensuring that observed radiation-associated microbial and molecular responses were not driven by a single donor or demographic subgroup. These details are now explicitly described in the Methods and summarized in Supplemental S1 Tables 1 and 2.

Revised methods: “To ensure that the coHSE model reflected representative human skin microbial diversity, colonization was performed using microbiome samples derived from 11 independent human donors, resulting in 20 total inoculations distributed across radiation dose groups (0 Gy: n = 8; 1 Gy: n = 6; 4 Gy: n = 6). Each experimental condition included microbiomes from multiple donors, and several donors contributed samples across different dose groups, reducing potential donor-specific bias. The donor cohort included both female (63.6%) and male (36.4%) participants and spanned two adult age ranges (20–40 years: 36.4%; 40–60 years: 63.6%). Although females were more represented overall and male donors were primarily represented in the younger age group, inclusion of multiple independent donors per condition was used to ensure that results were not driven by a single donor or demographic subgroup. This design captures natural inter-individual microbial variability and enhances the ecological validity of the coHSE model.”

- Please explain why 0 Gy, 1 Gy, and 4 Gy were selected and how these doses relate to real-world radiation exposure scenarios.

Response: The 1 Gy and 4 Gy doses were selected to represent exposure levels relevant to both experimental radiation biology and real-world exposure scenarios in the coHSE and mouse skin models. A dose of approximately 1 Gy falls within the range of individual fraction doses commonly used in radiotherapy (typically 1.8–2 Gy per fraction) and overlaps with exposure levels that may occur in occupational, accidental, or spaceflight contexts. A dose of 4 Gy corresponds to the cumulative effect of multiple radiotherapy fractions or localized acute exposures and extends into exposure levels associated with clinically biological effects, as recognized in radiation emergency response and biodosimetry frameworks, including IAEA and BARDA guidelines. Using this dose interval enabled evaluation of dose-dependent multi-omic responses and assessment of the ability of skin swab profiling to detect and discriminate molecular alterations across moderate and higher radiation exposure levels under realistic and translationally relevant photon exposure conditions. For clarity, we added this rational for dose selection in the introduction:

“Radiation doses of 1 Gy and 4 Gy were selected to reflect exposure levels relevant to fractionated radiotherapy and radiation emergency scenarios, spanning a biologically and clinically relevant range recognized in biodosimetry frameworks. This dose interval enables evaluation of dose-dependent molecular responses and assessment of the capacity of skin-based multi-omic profiling to detect and discriminate radiation exposure under realistic and translationally relevant photon exposure conditions.”

- Please elaborate on the rationale for using specific statistical approaches (e.g., DIABLO, sPLS-DA) and their limitations.

Response: We have expanded the Methods section to clarify both the rationale and limitations of using DIABLO and sPLS-DA. These methods were selected because they are specifically designed for supervised integration of multi-omic datasets measured on the same samples and enable identification of coordinated biomarker panels reflecting systemic biological responses to radiation exposure. This is particularly relevant for radiation biodosimetry, where molecular responses occur across multiple biological layers. We also explicitly acknowledge the limitations inherent to supervised approaches, including potential sensitivity to sample size and risk of overfitting. To mitigate these risks, we applied sparsity constraints, cross-validation-based model tuning, and confirmed findings using complementary statistical analyses and biological interpretation. These clarifications have now been added to the Methods section. Lines 330-395

- Please address how variability in microbial and metabolomic profiles was controlled or accounted for in the study.

Response: We expanded the Methods to list variability control steps across omics blocks, including standardized sampling (site/area), standardized timing, controlled shaving SOP, QC-based filtering, normalization strategies, and cross-validation. In addition, we now explicitly state n the Discussion that microbiome and metabolome are inherently variable, and this is one reason why multi-omic integration can improve robustness. Indeed, it is important to note that both the skin microbiome and metabolome exhibit substantial inter-individual and temporal variability, even under controlled conditions. This intrinsic biological variability reflects differences in host physiology, microbial composition, environmental exposure, and dynamic metabolic regulation. Rather than representing a limitation, this variability underscores the importance of integrative analytical approaches capable of identifying coordinated molecular responses across multiple biological layers. Multi-omic integration methods such as DIABLO leverage covariance structures across datasets to identify robust biomarker signatures that are consistently associated with radiation exposure despite individual variability within any single omic layer. By focusing on correlated, cross-domain molecular patterns rather than isolated features, this approach enhances robustness and improves the reliability of identifying biologically meaningful radiation-associated signatures.

Added paragraph in the Discussion: “The skin microbiome and metabolome are inherently variable across individuals and time, reflecting dynamic host–microbe interactions and metabolic regulation. Multi-omic integration approaches such as DIABLO help address this variability by identifying coordinated molecular signatures across datasets, thereby improving robustness and reliability compared with single-omic analyses.” Lines 829-836


Discussion

- Please provide more details on how the findings can be applied to human biodosimetry and the steps needed to develop practical tools for real-time monitoring.

Response: We added a subsection outlining key translational steps: (1) validation in human cohorts (e.g., radiotherapy patients with known dose), (2) evaluation of confounders (site, sex, age, skincare products, antibiotics), (3) model calibration and thresholding, (4) simplified assay development (targeted panels), and (5) development of operational workflows for rapid processing. We also clarified that “real-time” is a future direction requiring technology integration and prospective testing:

“To translate these findings into practical human biodosimetry tools, several steps are required: (1) validation in human cohorts with known exposures (e.g., radiotherapy patients with defined fractionated doses); (2) systematic evaluation of key confounders (anatomical site, sex, age, hygiene/skincare products, antibiotics/medications, and health status); (3) model calibration, including definition of classification thresholds and assessment of temporal stability; (4) simplified assay development using targeted biomarker panels derived from the discovery signatures; and (5) establishment of operational workflows enabling standardized sampling, rapid processing, and scalable deployment. (6) Finally, emerging transdermal sensing technologies provide a realistic pathway toward longitudinal and potentially real-time monitoring: non-invasive Raman spectroscopy can detect molecular fingerprints directly through intact skin including metabolites such as glucose, urea, and uric acid, while wearable microneedle-based sensors enable minimally invasive, continuous measurement of biochemical markers in interstitial fluid (5,6). Together, these advances support future integration of validated radiation-responsive biomarkers into wearable biodosimetry platforms that could complement existing cytogenetic and blood-based approaches.” Lines 866-880

- Please further explain how microbial shifts contribute to skin recovery and systemic health, particularly in the context of radiation exposure.

Response: We thank the reviewer for this insightful suggestion, which helped us clarify the biological interpretation of the microbial shifts observed in this study. Accordingly, we now discuss (1) the enrichment of SCFA-producing taxa such as *Lachnospiraceae* and *Lactobacillales* and their known roles in producing metabolites (e.g., butyrate and propionate) that contribute to epithelial homeostasis; (2) potential implications for skin barrier repair mechanisms, including links with natural moisturizing factor–related metabolites and lipid remodeling pathways involved in epidermal recovery after radiation-induced stress; and (3) potential systemic relevance, highlighting emerging evidence for skin–microbiome–host interactions analogous to the gut–skin axis and the possible association of several identified metabolites with cardiovascular and inflammatory pathways. Lines 718-769

- Please discuss the need for future studies to investigate a wider range of radiation doses to improve generalizability.

Response: We agree that evaluating a broader range of radiation doses would improve generalizability. We have added a statement in the Discussion noting that future studies should examine additional dose levels and exposure scenarios to better characterize dose–response relationships and refine skin-based biodosimetry signatures.

- Please provide more evidence or references to support the hypothesis that skin responses may reflect systemic health, including cardiovascular injury.

Response: We added additional citations supporting the concept that skin can reflect systemic physiology via circulating metabolites, inflammatory mediators, and barrier-associated lipids. Importantly, we also revised the text to clearly label these as hypotheses and not conclusions drawn from this dataset, since systemic outcomes were not measured:

“Of note, cutaneous manifestations typical of radiation exposure, such as edema, telangiectasia, and ulceration, also commonly appear in various cardiovascular diseases, particularly those with inflammatory components (109–113). The skin, involved in thermoregulation, vascular tone, and immunity, receives ~10% of cardiac output, making it biologically relevant for reflecting systemic injury signals. Interestingly, several discriminating metabolites identified here (e.g., phenylalanine, 3-nitrotyrosine, hypoxanthine, succinate, and trimethyllysine) have also been implicated in cardiovascular dysfunction (106–108). Others, such as acylcarnitines, inosine, xanthine, and taurine, correlate with myocardial ischemia, remodeling, or heart failure progression (114–116). Interestingly, skin levels of metabolic markers such as cholesterol and carotenoids have been associated with cardiovascular disease risk (117,118). These observations suggest that skin alterations reflect broader systemic conditions and might serve as a non-invasive window to assess internal damage. Supporting this concept, optical skin readouts have been shown to predict cardiovascular disease and mortality risk in both murine models and human cohorts (119–121). Further studies, including targeted imaging and histopathology, are needed to confirm associations between omic profiles and radiation-induced injury.” Lines 796-818

- Please clarify the functional significance of lipid remodeling and its potential as a biomarker for radiation exposure.

Response: As suggested, we have expanded the Discussion to better explain the functional significance of lipid remodeling observed after radiation exposure. Specifically, we now discuss (1) the role of lipid remodeling in epidermal barrier maintenance, including compensatory changes in fatty acid synthesis and structural lipids that contribute to restoration of the stratum corneum permeability barrier; (2) the involvement of signaling lipids such as DAG and phosphatidic acid in cellular stress responses, including pathways linked to inflammation, proliferation, and tissue repair; and (3) the potential value of these lipid alterations as biodosimetric indicators, as several discriminant lipid species and lipid network perturbations showed dose-dependent patterns across both experimental models. Lines 741-769

- Please discuss the functional roles of the identified microbial taxa and their potential contributions to skin recovery and systemic health.

Response: Please see response for “- Please further explain how microbial shifts contribute to skin recovery and systemic health, particularly in the context of radiation exposure.”

Conclusion

Highlight limitations: Summarize the key limitations of the study and suggest specific areas for future research.

Response: We have now expanded the Discussion to explicitly summarize the key limitations of the study and outline specific future research directions. In particular, we acknowledge the modest sample size and the exploratory nature of the multi-omic biomarker discovery approach, which resulted in moderate and variable cross-validation performance and highlights the need for validation in larger independent cohorts. We also discuss demographic imbalance in donor-derived microbiome samples and note the importance of evaluating broader population variability in future studies. In addition, we clarify methodological limitations inherent to supervised integrative modeling approaches and emphasize that the identified signatures should be interpreted as candidate biomarkers requiring further validation.

Expand on practical applications: Provide more details on how the findings can be translated into real-world biodosimetry tools, including the use of emerging technologies like Raman or infrared spectroscopy.

Response: We believe the paragraph added in the discussion previously mentioned in comment “- Please provide more details on how the findings can be applied to human biodosimetry and the steps needed to develop practical tools for real-time monitoring.” , also address the present reviewer comment.

Acknowledgements

Clarify contributions: Provide more details on the specific roles of each author in the study.

Response: The Author Contributions section has been expanded to provide a clearer description of the specific roles of each author using standard contribution categories (e.g., conceptualization, methodology, investigation, data curation, formal analysis, writing, supervision, and funding acquisition).

Contributions:

Conceptualization: KA, ECL, GG. Methodology: GV, KA, ECL, GG, DCL, BC. Investigation: GV, JA, JF, BP, JLI, AMS, JHM, SH. Data Curation: GV, SIG, PA, DCV, PI. Formal Analysis: GV, MAS. Resources: KA, ECL, GG, DCL, BC. Visualization: GV. Writing (Original Draft): GV. Writing (Review & Editing): KA, ECL, GG, GV, JA, JF, BP, JLI, AMS, JHM, SH, SIG, PA, DCV, PI, MAS, DCL, BC. Supervision: KA, ECL, GG. Funding Acquisition: KA, ECL

Description:

GV performed the multi-omic data processing and analysis, including untargeted metabolomics, targeted lipidomics, and 16S microbiome integration. SIG, PA, DCV, and PI processed and curated the 16S sequencing datasets. DCL and BC provided training and expertise for the colonized human skin equivalent (coHSE) model.

Expand on funding sources: Clarify the role of funders in the study design, data collection, analysis, and publication decisions.

Response: The Acknowledgements section specifies the funding sources supporting the work, including the IARPA TEI-REX program through the Army Research Office and the NCI P30 Cancer Center Support Grant. These funding statements also include the standard disclaimer that the views expressed are those of the authors and do not necessarily reflect the official policies of the funding agencies. To further clarify this point, we have now explicitly stated that the funders had no role in the study design, data collection, data analysis, interpretation of results, manuscript preparation, or publication decisions.

References

Ensure completeness: Verify that all references are properly cited and formatted.
Highlight key references: Identify and emphasize the most relevant references that support the study’s findings and conclusions.


Supporting Information

Provide more details: Expand on the supplementary figures and tables to ensure clarity and accessibility for readers.

Response: We revised the descriptions of the supplementary figures and tables and clarified their relevance to the main findings of the study:

**S1 File.** Supplementary information file containing additional methodological details and extended datasets supporting the analyses presented in the main manuscript.

**S1 Fig.** Pathway enrichment analysis of metabolites differentially altered between 1 Gy and 4 Gy exposed coHSE samples (ORA, KEGG library), highlighting metabolic pathways contributing to radiation dose discrimination.

**S2 Fig.** Spectral abundance profiles of radio-responsive metabolites in coHSE samples, supporting identification of conserved metabolic signatures associated with radiation exposure.

**S3 Fig.** Spectral abundance of natural moisturizing factor (NMF) components in coHSE following radiation exposure, illustrating alterations in metabolites associated with epidermal barrier homeostasis.

**S4 Fig.** Lipid reaction network analysis showing suppressed and activated lipid transformations in coHSE following radiation exposure, highlighting remodeling of lipid metabolic pathways.

**S5 Fig.** Diagnostic plots of DIABLO multiblock sPLS-DA model performance for the second latent component (component 2) in mouse skin following radiation exposure.

**S6 Fig.** Lipid reaction network analysis showing suppressed and activated lipid transformations in mouse skin following radiation exposure.

**S7 Fig.** Receiver operating characteristic (ROC) curve analysis evaluating the performance of NMF metabolites and candidate biomarker panels for discriminating non-exposed (0 Gy) versus exposed (1 Gy and 4 Gy) samples, and for distinguishing 1 Gy from 4 Gy exposure.

**Supplementary Tables**

**S1 Tables.** A) Human donor microbiome diversity used for coHSE inoculation, showing the distribution of samples contributed by each donor across radiation exposure groups; B) Demographic distribution of microbiome donors (sex and age ranges) contributing to the coHSE inoculation.

**S2 Table.** MS/MS fragment matches used for metabolite identification in mouse skin swab samples. Metabolites were annotated based on matches to pure standards (Metabolomics Standards Initiative Level 1) or spectral libraries (HMDB and METLIN; Level 2). Unmatched fragments are indicated by “//”.

**S3 Table.** Differentially abundant microbial taxa (Cluster 1) identified in mouse skin following 1 Gy and 4 Gy radiation exposure.

Explain the significance: Clarify the importance of the supplementary data in supporting the main findings of the study.

Response: Supplementary figures provide additional analytical detail supporting the multi-omic integration results, including pathway enrichment analyses, metabolite spectral abundance profiles, lipid reaction network perturbations, and diagnostic plots of the DIABLO multiblock sPLS-DA models. These figures allow readers to evaluate the robustness of the metabolic and lipidomic patterns underlying the main results presented in the manuscript. The supplementary tables provide detailed methodological and biological context supporting the experimental design and data interpretation. For example, S1 Tables document the diversity and demographic distribution of human donors used for microbiome inoculation of the coHSE model, demonstrating that multiple independent donors contributed to each radiation condition and capturing natural inter-individual microbiome variability. S2 Table provides metabolite identification confidence levels and MS/MS spectral matches according to Metabolomics Standards Initiative criteria, ensuring transparency of metabolite annotation. S3 Table reports the differentially abundant microbial taxa identified in the mouse skin model.
