# Supplementary material for: Monitoring radiation exposure through skin swab multi-omic profiling
Source: PLoS One. 2026 Aug 5;21(8):e0354734. doi: 10.1371/journal.pone.0354734 (PMC13440882; doi:10.1371/journal.pone.0354734)
Supplement: Peer review history

# Peer review: Decision Letter 0

**DOI:** 10.1371/journal.pone.0354734.r001

**Authors:** Yi Cao

14 Jan 2026

PONE-D-25-63496

Monitoring radiation exposure through skin swab multi-omic profiling

PLOS One

Dear Dr. Laiakis,

Thank you for submitting your manuscript to PLOS ONE. After careful consideration, we feel that it has merit but does not fully meet PLOS ONE’s publication criteria as it currently stands. Therefore, we invite you to submit a revised version of the manuscript that addresses the points raised during the review process.

Please submit your revised manuscript by Feb 28 2026 11:59PM. If you will need more time than this to complete your revisions, please reply to this message or contact the journal office at plosone@plos.org. When you're ready to submit your revision, log on to https://www.editorialmanager.com/pone/ and select the 'Submissions Needing Revision' folder to locate your manuscript file.

Please include the following items when submitting your revised manuscript:

- A letter that responds to each point raised by the academic editor and reviewer(s). You should upload this letter as a separate file labeled 'Response to Reviewers'.
- A marked-up copy of your manuscript that highlights changes made to the original version. You should upload this as a separate file labeled 'Revised Manuscript with Track Changes'.
- An unmarked version of your revised paper without tracked changes. You should upload this as a separate file labeled 'Manuscript'.

If you would like to make changes to your financial disclosure, please include your updated statement in your cover letter. Guidelines for resubmitting your figure files are available below the reviewer comments at the end of this letter.

If applicable, we recommend that you deposit your laboratory protocols in protocols.io to enhance the reproducibility of your results. Protocols.io assigns your protocol its own identifier (DOI) so that it can be cited independently in the future. For instructions see: https://journals.plos.org/plosone/s/submission-guidelines#loc-laboratory-protocols. Additionally, PLOS ONE offers an option for publishing peer-reviewed Lab Protocol articles, which describe protocols hosted on protocols.io. Read more information on sharing protocols at https://plos.org/protocols?utm_medium=editorial-email&utm_source=authorletters&utm_campaign=protocols.

We look forward to receiving your revised manuscript.

Kind regards,

Yi Cao

Academic Editor

PLOS One

**Journal Requirements:**

1. When submitting your revision, we need you to address these additional requirements.

Please ensure that your manuscript meets PLOS ONE's style requirements, including those for file naming. The PLOS ONE style templates can be found at

https://journals.plos.org/plosone/s/file?id=wjVg/PLOSOne_formatting_sample_main_body.pdf and

https://journals.plos.org/plosone/s/file?id=ba62/PLOSOne_formatting_sample_title_authors_affiliations.pdf

2. To comply with PLOS One submissions requirements, in your Methods section, please provide additional information regarding the experiments involving animals and ensure you have included details on (a) methods of sacrifice, (b) methods of anesthesia and/or analgesia, and (c) efforts to alleviate suffering.

3. Thank you for stating in your Funding Statement:

This research was supported by the Intelligence Advanced Research Projects Activity (IARPA) TEI-REX program through the Army Research Office contract No.W911NF-22-C-0064 (P.I. Karyn Apfeldorf). Animal procedures realized at Lawrence Berkeley National Laboratory were supported by the IARPA TEI-REX contract number 20008-D2021-2107310008. The views and conclusions contained should not be interpreted as necessarily representing the official policies, either expressed or implied, of ODNI, IARPA, ARO, or the U.S. Government. The U.S. Government is authorized to reproduce and distribute reprints for governmental purposes notwithstanding any copyright annotation therein. The project described above was also supported by award no. P30 CA051008 (P.I. Louis Weiner) from the National Cancer Institute (NCI). The content is solely the responsibility of the authors and does not necessarily represent the official views of the NCI or the NIH.

Please provide an amended statement that declares *all* the funding or sources of support (whether external or internal to your organization) received during this study, as detailed online in our guide for authors at http://journals.plos.org/plosone/s/submit-now.  Please also include the statement “There was no additional external funding received for this study.” in your updated Funding Statement.

Please include your amended Funding Statement within your cover letter. We will change the online submission form on your behalf.

4. Thank you for stating the following financial disclosure:

This research was supported by the Intelligence Advanced Research Projects Activity (IARPA) TEI-REX program through the Army Research Office contract No.W911NF-22-C-0064 (P.I. Karyn Apfeldorf). Animal procedures realized at Lawrence Berkeley National Laboratory were supported by the IARPA TEI-REX contract number 20008-D2021-2107310008. The views and conclusions contained should not be interpreted as necessarily representing the official policies, either expressed or implied, of ODNI, IARPA, ARO, or the U.S. Government. The U.S. Government is authorized to reproduce and distribute reprints for governmental purposes notwithstanding any copyright annotation therein. The project described above was also supported by award no. P30 CA051008 (P.I. Louis Weiner) from the National Cancer Institute (NCI). The content is solely the responsibility of the authors and does not necessarily represent the official views of the NCI or the NIH.

Please state what role the funders took in the study.  If the funders had no role, please state: "The funders had no role in study design, data collection and analysis, decision to publish, or preparation of the manuscript."

If this statement is not correct you must amend it as needed.

Please include this amended Role of Funder statement in your cover letter; we will change the online submission form on your behalf.

5. Thank you for stating the following in the Acknowledgments Section of your manuscript:

This research was supported by the Intelligence Advanced Research Projects Activity (IARPA) TEI-REX program through the Army Research Office contract No.W911NF-22-C-0064 (P.I. Karyn Apfeldorf). Animal procedures realized at Lawrence Berkeley National Laboratory were supported by the IARPA TEI-REX contract number 20008-D2021-2107310008. The views and conclusions contained should not be interpreted as necessarily representing the official policies, either expressed or implied, of ODNI, IARPA, ARO, or the U.S. Government. The U.S. Government is authorized to reproduce and distribute reprints for governmental purposes notwithstanding any copyright annotation therein. The project described above was also supported by award no. P30 CA051008 (P.I. Louis Weiner) from the National Cancer Institute (NCI). The content is solely the responsibility of the authors and does not necessarily represent the official views of the NCI or the NIH. Raw 16 S rRNA sequencing data have been deposited in the NCBI Sequence Read Archive under accession number [PRJNA1308618] and are available at < https://www.ncbi.nlm.nih.gov/bioproject/1308618>. All other data supporting the findings are available from the corresponding author upon reasonable request.

We would also like to thank Paul Peshette for his support to team members from Arete Associates.

We note that you have provided funding information that is not currently declared in your Funding Statement. However, funding information should not appear in the Acknowledgments section or other areas of your manuscript. We will only publish funding information present in the Funding Statement section of the online submission form.

Please remove any funding-related text from the manuscript and let us know how you would like to update your Funding Statement. Currently, your Funding Statement reads as follows:

This research was supported by the Intelligence Advanced Research Projects Activity (IARPA) TEI-REX program through the Army Research Office contract No.W911NF-22-C-0064 (P.I. Karyn Apfeldorf). Animal procedures realized at Lawrence Berkeley National Laboratory were supported by the IARPA TEI-REX contract number 20008-D2021-2107310008. The views and conclusions contained should not be interpreted as necessarily representing the official policies, either expressed or implied, of ODNI, IARPA, ARO, or the U.S. Government. The U.S. Government is authorized to reproduce and distribute reprints for governmental purposes notwithstanding any copyright annotation therein. The project described above was also supported by award no. P30 CA051008 (P.I. Louis Weiner) from the National Cancer Institute (NCI). The content is solely the responsibility of the authors and does not necessarily represent the official views of the NCI or the NIH.

Please include your amended statements within your cover letter; we will change the online submission form on your behalf.

6. In the online submission form, you indicated that “All other data supporting the findings are available from the corresponding author upon reasonable request.”

All PLOS journals now require all data underlying the findings described in their manuscript to be freely available to other researchers, either a. In a public repository, b. Within the manuscript itself, or c. Uploaded as supplementary information.

This policy applies to all data except where public deposition would breach compliance with the protocol approved by your research ethics board. If your data cannot be made publicly available for ethical or legal reasons (e.g., public availability would compromise patient privacy), please explain your reasons on resubmission and your exemption request will be escalated for approval.

7. Please upload a new copy of Figures 1 – 8, S1, S4, S5, S6 and S7, as the detail is not clear. Please follow the link for more information:  https://journals.plos.org/plosone/s/figures

8. Please include a new copy of Tables S1 and S2 in your manuscript; the current table is difficult to read. Please follow the link for more information: https://journals.plos.org/plosone/s/tables

9. If the reviewer comments include a recommendation to cite specific previously published works, please review and evaluate these publications to determine whether they are relevant and should be cited. There is no requirement to cite these works unless the editor has indicated otherwise.

[Note: HTML markup is below. Please do not edit.]

Reviewers' comments:

Reviewer's Responses to Questions

**Comments to the Author**

1. Is the manuscript technically sound, and do the data support the conclusions?

The manuscript must describe a technically sound piece of scientific research with data that supports the conclusions. Experiments must have been conducted rigorously, with appropriate controls, replication, and sample sizes. The conclusions must be drawn appropriately based on the data presented.

Reviewer #1: Yes

Reviewer #2: Partly

Reviewer #3: Yes

**********

2. Has the statistical analysis been performed appropriately and rigorously?

Reviewer #1: Yes

Reviewer #2: Yes

Reviewer #3: Yes

**********

3. Have the authors made all data underlying the findings in their manuscript fully available?

The PLOS Data policy requires authors to make all data underlying the findings described in their manuscript fully available without restriction, with rare exception (please refer to the Data Availability Statement in the manuscript PDF file). The data should be provided as part of the manuscript or its supporting information, or deposited to a public repository. For example, in addition to summary statistics, the data points behind means, medians and variance measures should be available. If there are restrictions on publicly sharing data—e.g. participant privacy or use of data from a third party—those must be specified.

Reviewer #1: Yes

Reviewer #2: Yes

Reviewer #3: Yes

**********

4. Is the manuscript presented in an intelligible fashion and written in standard English?

PLOS ONE does not copyedit accepted manuscripts, so the language in submitted articles must be clear, correct, and unambiguous. Any typographical or grammatical errors should be corrected at revision, so please note any specific errors here.

Reviewer #1: Yes

Reviewer #2: Yes

Reviewer #3: Yes

**********

5. Review Comments to the Author

Please use the space provided to explain your answers to the questions above. You may also include additional comments for the author, including concerns about dual publication, research ethics, or publication ethics. (Please upload your review as an attachment if it exceeds 20,000 characters)

**Reviewer #1:**  Summary

This manuscript presents a multi-omic study proposing skin swab–based metabolomic, lipidomic, and microbiome profiling as a non-invasive biodosimetry platform for ionizing radiation exposure. Using colonized human skin equivalents and mouse models exposed to 0, 1, or 4 Gy of x-rays, the authors apply DIABLO-based multiblock sPLS-DA to identify correlated, dose-responsive molecular and microbial signatures. While the experimental execution is extensive and the datasets are large, the study ultimately prioritizes multivariate discrimination over biological rigor. Many conclusions rely heavily on supervised classification, post-hoc feature selection, and pathway over-interpretation, without sufficient control for overfitting, temporal dependence, or biological confounding. As a result, the manuscript reads more as a methodological showcase than a validated biodosimetry framework, and its translational claims are substantially overstated.

Strengths

The study addresses a relevant and timely problem in radiation biology: the need for rapid, non-invasive biodosimetry. The use of skin swabs is innovative and practically attractive, and the parallel analysis of human skin equivalents and mouse skin is conceptually sound. The experimental workflows for metabolomics, lipidomics, and 16S profiling are technically competent and well described. The authors make effective use of modern multi-omic integration tools and present a large amount of data in a transparent manner. The inclusion of ROC analyses and dose-resolved comparisons demonstrates an effort to evaluate diagnostic performance rather than relying solely on descriptive trends.

Recommendations

The central weakness of the manuscript lies in its analytical strategy. The study relies almost exclusively on supervised sPLS-DA (DIABLO) to define “radiation-responsive” features, yet provides insufficient safeguards against overfitting.

Sample sizes per group are small relative to the number of features, time points are pooled rather than modeled explicitly, and the same datasets are repeatedly used for feature selection and performance evaluation.

High AUC values reported for refined metabolite panels, particularly those approaching 0.99, are therefore not convincing without independent validation, permutation testing, or nested cross-validation. At minimum, the authors should demonstrate model robustness through repeated resampling and clearly separate training and evaluation steps.

Temporal structure is inadequately handled. Samples collected at 1, 3, and 7 days post-irradiation are pooled for many analyses, despite the well-known time dependence of radiation responses.

Pooling obscures whether identified signatures represent true dose effects or simply reflect differences in recovery kinetics. A proper longitudinal analysis, or at least stratified presentation by time point, is necessary to support claims of dose-specific signatures.

Biological interpretation consistently overreaches the data. Pathway enrichment results are derived from putatively identified metabolites selected via supervised models, yet are discussed as evidence of coordinated biological programs (e.g., barrier disruption, mitochondrial dysfunction, systemic cardiovascular risk).

These interpretations are speculative in the absence of orthogonal validation such as histology, barrier function assays, inflammatory readouts, or targeted biochemical measurements. The frequent extension of skin molecular changes to systemic health implications is particularly problematic given that no systemic endpoints were measured.

Microbiome analyses raise similar concerns. Changes in taxa such as Lachnospiraceae and Lactobacillales are repeatedly framed as radioprotective or reparative, but the study design cannot distinguish direct radiation effects from secondary ecological shifts or housing effects. Moreover, 16S-based taxonomic resolution is insufficient to support functional claims without complementary metagenomic or metabolomic corroboration.

While the integration of multi-omic skin swab data is technically impressive, the study does not yet establish a deployable biodosimetry tool. Claims regarding real-time monitoring, emergency response utility, or spaceflight applications are premature and should be clearly labeled as future directions rather than inferred outcomes.

Conclusion

This is a data-rich and technically sophisticated study, but it is analytically and interpretively overextended. The reliance on supervised multivariate methods without adequate validation, the pooling of temporally distinct samples, and the speculative biological extrapolations substantially weaken the manuscript’s conclusions. As currently presented, the work does not convincingly demonstrate a robust, generalizable skin-based biodosimetry platform. Major restructuring of the analysis, substantial tempering of claims, and additional validation would be required to support publication.

**Reviewer #2:** This study systematically investigates the dose-dependent response of skin to ionizing radiation by integrating multi-omics (microbiome, metabolome, lipidome) and cross-model (human skin equivalent coHSE, mouse) approaches. The research design is novel, and the data volume is substantial. It is the first to propose a "host-microbe co-metabolism" framework for radiation response at the skin level and to screen biomarker combinations with high diagnostic potential, which holds significant value for the development of radiation biodosimetry. However, the paper has deficiencies in the rigor of experimental details, the depth of data interpretation, and logical coherence, requiring substantial revisions to support its important conclusions.

1.P150: Briefly explain why the control group has a larger sample size.

2.Briefly state the rationale for selecting doses of 1 Gy and 4 Gy.

3.Shaving itself is a strong physical and mildly traumatic stimulus that can significantly disrupt the skin barrier, alter the local microenvironment (e.g., temperature, humidity), and trigger skin inflammation and repair responses. This greatly perturbs the structure of the skin microbial community before sampling, making it unrepresentative of the "steady-state" skin flora, instead reflecting a "post-perturbation recovery" state. The animal experiment design of "shaving 24 hours before sampling" severely interferes with skin microbial homeostasis. This constitutes a fundamental flaw that must be modified or provide strong reasons.

4.The temporal design alignment between the human skin equivalent (coHSE) and mouse experiments is excellent, and the use of the same irradiation device is a major advantage. However, the inconsistency in key irradiation physical parameters (energy, filter, dose rate) means the radiation received in the two experiments differs in physical characteristics. The rationale for selecting these parameters should be briefly explained.

5.The current statistical description is the weakest part of the methods section. It fails to match the complex time-dose multi-factorial design of your study. It is recommended to rewrite the statistics section with a more rigorous and detailed description.

6.P445-447: It is suggested to change to: "1 Gy radiation exposure was primarily associated with the activation of carbohydrate metabolism pathways, while concurrent changes in the abundance of microbial taxa such as Lachnospiraceae were observed."

7.P474: Confirm the accuracy of the lipid nomenclature for "TAG(52:7)-FA(16:0), and (-2)Cl66:3-FA(16:1)".

8.P500: Change "1Gy" to "4Gy".

9.Much of the Discussion section reiterates specific observations already detailed in the Results, without sufficient interpretation, synthesis, or attribution of new meaning. The Discussion should avoid being a secondary narration of the results. It is recommended to significantly condense the restatement of specific results and instead emphasize the new concepts, models, or insights distilled from these results.

**Reviewer #3:** Overall comment:

This study demonstrated that radiation caused dose-dependent shifts in the skin microbiome in both human skin equivalents and mice. Several taxa - especially Lachnospiraceae/Oscillospiraceae/Bacillaceae - emerged as candidate biomarkers for non-invasive radiation biodosimetry. Although the study demonstrates the potential of skin swab multi-omic profiling for biodosimetry, the translational application of these findings to human settings remains uncertain. Additional research is needed to validate the findings in human subjects and develop practical tools for real-time monitoring. The study uses relatively small sample sizes for both coHSEs and mouse models, which may limit the statistical power and generalizability of the results. Additionally, the study pools microbial samples from human donors to inoculate coHSEs, which may not fully replicate the diversity and complexity of individual human skin microbiomes.

Specific comments

Abstract

- Please provide more details on sample sizes of human skin equivalents and murine models and how the findings can be applied to human biodosimetry.

Introduction

- Please provide more detailed comparisons of existing biodosimetry methods and their shortcomings.

- Please 3xplain why skin swabs are particularly suitable for biodosimetry compared to other non-invasive methods.

- Please highlight the need for validation in human subjects and the challenges of translating findings from animal models and coHSEs.

Materials and Methods

- Please provide more details on the diversity of microbial samples used to inoculate coHSEs and how representative they are of human skin microbiomes.

- Please explain why 0 Gy, 1 Gy, and 4 Gy were selected and how these doses relate to real-world radiation exposure scenarios.

- Please elaborate on the rationale for using specific statistical approaches (e.g., DIABLO, sPLS-DA) and their limitations.

- Please address how variability in microbial and metabolomic profiles was controlled or accounted for in the study.

Results

- Please provide more detailed explanations of the biological significance of dose-specific microbial and metabolomic changes.

- Please provide more clarity on the implications of lipid remodeling and its connection to skin barrier function and radiation injury.

- Please address how variability in responses at higher doses (4 Gy) might affect the reliability of biodosimetry.

Discussion

- Please provide more details on how the findings can be applied to human biodosimetry and the steps needed to develop practical tools for real-time monitoring.

- Please further explain how microbial shifts contribute to skin recovery and systemic health, particularly in the context of radiation exposure.

- Please discuss the need for future studies to investigate a wider range of radiation doses to improve generalizability.

- Please provide more evidence or references to support the hypothesis that skin responses may reflect systemic health, including cardiovascular injury.

- Please clarify the functional significance of lipid remodeling and its potential as a biomarker for radiation exposure.

- Please discuss the functional roles of the identified microbial taxa and their potential contributions to skin recovery and systemic health.

Conclusion

Highlight limitations: Summarize the key limitations of the study and suggest specific areas for future research.

Expand on practical applications: Provide more details on how the findings can be translated into real-world biodosimetry tools, including the use of emerging technologies like Raman or infrared spectroscopy.

Acknowledgements

Clarify contributions: Provide more details on the specific roles of each author in the study.

Expand on funding sources: Clarify the role of funders in the study design, data collection, analysis, and publication decisions.

References

Ensure completeness: Verify that all references are properly cited and formatted.

Highlight key references: Identify and emphasize the most relevant references that support the study’s findings and conclusions.

Supporting Information

Provide more details: Expand on the supplementary figures and tables to ensure clarity and accessibility for readers.

Explain the significance: Clarify the importance of the supplementary data in supporting the main findings of the study.

By addressing these points, the study can improve its clarity, robustness, and potential for real-world application.

**********

6. PLOS authors have the option to publish the peer review history of their article (what does this mean?). If published, this will include your full peer review and any attached files.

If you choose “no”, your identity will remain anonymous but your review may still be made public.

**Do you want your identity to be public for this peer review?**  For information about this choice, including consent withdrawal, please see our Privacy Policy.

Reviewer #1: No

Reviewer #2: No

Reviewer #3: No

**********

[NOTE: If reviewer comments were submitted as an attachment file, they will be attached to this email and accessible via the submission site. Please log into your account, locate the manuscript record, and check for the action link "View Attachments". If this link does not appear, there are no attachment files.]

To ensure your figures meet our technical requirements, please review our figure guidelines: https://journals.plos.org/plosone/s/figures

You may also use PLOS’s free figure tool, NAAS, to help you prepare publication quality figures: https://journals.plos.org/plosone/s/figures#loc-tools-for-figure-preparation.

NAAS will assess whether your figures meet our technical requirements by comparing each figure against our figure specifications.

# Author comment: Author response to Decision Letter 1

**DOI:** 10.1371/journal.pone.0354734.r002

20 May 2026

We have upload a point-by-point response to the reviewers' comments in a separate file.

Attachment Submitted filename: PLOSone rebuttal 052026.docx

# Peer review: Decision Letter 1

**DOI:** 10.1371/journal.pone.0354734.r003

**Authors:** Yi Cao

13 Jul 2026

Monitoring radiation exposure through skin swab multi-omic profiling

PONE-D-25-63496R1

Dear Dr. Laiakis,

We’re pleased to inform you that your manuscript has been judged scientifically suitable for publication and will be formally accepted for publication once it meets all outstanding technical requirements.

Within one week, you’ll receive an e-mail detailing the required amendments. When these have been addressed, you’ll receive a formal acceptance letter and your manuscript will be scheduled for publication.

An invoice will be generated when your article is formally accepted. Please note, if your institution has a publishing partnership with PLOS and your article meets the relevant criteria, all or part of your publication costs will be covered. Please make sure your user information is up-to-date by logging into Editorial Manager at Editorial Manager® and clicking the ‘Update My Information' link at the top of the page. For questions related to billing, please contact billing support.

If your institution or institutions have a press office, please notify them about your upcoming paper to help maximize its impact. If they’ll be preparing press materials, please inform our press team as soon as possible -- no later than 48 hours after receiving the formal acceptance. Your manuscript will remain under strict press embargo until 2 pm Eastern Time on the date of publication. For more information, please contact onepress@plos.org.

Kind regards,

Yi Cao

Academic Editor

PLOS One

Additional Editor Comments (optional):

Reviewers' comments:

Reviewer's Responses to Questions

**Comments to the Author**

1. If the authors have adequately addressed your comments raised in a previous round of review and you feel that this manuscript is now acceptable for publication, you may indicate that here to bypass the “Comments to the Author” section, enter your conflict of interest statement in the “Confidential to Editor” section, and submit your "Accept" recommendation.

Reviewer #1: All comments have been addressed

Reviewer #2: All comments have been addressed

**********

2. Is the manuscript technically sound, and do the data support the conclusions?<br/><br/>The manuscript must describe a technically sound piece of scientific research with data that supports the conclusions. Experiments must have been conducted rigorously, with appropriate controls, replication, and sample sizes. The conclusions must be drawn appropriately based on the data presented.

Reviewer #1: Yes

Reviewer #2: Yes

**********

3. Has the statistical analysis been performed appropriately and rigorously?

Reviewer #1: Yes

Reviewer #2: Yes

**********

4. Have the authors made all data underlying the findings in their manuscript fully available?<br/><br/>The PLOS Data policy requires authors to make all data underlying the findings described in their manuscript fully available without restriction, with rare exception (please refer to the Data Availability Statement in the manuscript PDF file). The data should be provided as part of the manuscript or its supporting information, or deposited to a public repository. For example, in addition to summary statistics, the data points behind means, medians and variance measures should be available. If there are restrictions on publicly sharing data—e.g. participant privacy or use of data from a third party—those must be specified.

Reviewer #1: Yes

Reviewer #2: Yes

**********

5. Is the manuscript presented in an intelligible fashion and written in standard English?<br/><br/>PLOS ONE does not copyedit accepted manuscripts, so the language in submitted articles must be clear, correct, and unambiguous. Any typographical or grammatical errors should be corrected at revision, so please note any specific errors here.

Reviewer #1: Yes

Reviewer #2: Yes

**********

6. Review Comments to the Author<br/><br/>Please use the space provided to explain your answers to the questions above. You may also include additional comments for the author, including concerns about dual publication, research ethics, or publication ethics. (Please upload your review as an attachment if it exceeds 20,000 characters)

Reviewer #1: The revision addresses the major concerns raised during review. The authors have clarified the exploratory role of the DIABLO analysis, added independent validation of the metabolite panel, included cross-validation and permutation testing, and toned down the interpretation of the AUC results. They also provide a clearer rationale for pooling early time points, dose selection, shaving procedures, irradiation parameters, donor microbiome diversity, and statistical methods. Importantly, the biological and translational claims are now more balanced, with microbiome shifts, pathway enrichment, systemic implications, and real-time biodosimetry applications presented as hypotheses or future directions rather than established conclusions. Overall, the manuscript is much improved, the remaining limitations are clearly acknowledged, and I have no further major concerns. I consider the manuscript ready for publication.

Reviewer #2: The author has already answered all the questions I raised.I have no further comments. I recommend publishing forPLOS One.

**********

7. PLOS authors have the option to publish the peer review history of their article (what does this mean?). If published, this will include your full peer review and any attached files.

If you choose “no”, your identity will remain anonymous but your review may still be made public.

**Do you want your identity to be public for this peer review?**  For information about this choice, including consent withdrawal, please see our Privacy Policy.

Reviewer #1: No

Reviewer #2: No

**********

# Editor report: Acceptance letter

**DOI:** 10.1371/journal.pone.0354734.r004

**Authors:** Yi Cao

PONE-D-25-63496R1

PLOS One

Dear Dr. Laiakis,

I'm pleased to inform you that your manuscript has been deemed suitable for publication in PLOS One. Congratulations! Your manuscript is now being handed over to our production team.

At this stage, our production department will prepare your paper for publication. This includes ensuring the following:

* All references, tables, and figures are properly cited

* All relevant supporting information is included in the manuscript submission,

* There are no issues that prevent the paper from being properly typeset

You will receive further instructions from the production team, including instructions on how to review your proof when it is ready. Please keep in mind that we are working through a large volume of accepted articles, so please give us a few days to review your paper and let you know the next and final steps.

Lastly, if your institution or institutions have a press office, please let them know about your upcoming paper now to help maximize its impact. If they'll be preparing press materials, please inform our press team within the next 48 hours. Your manuscript will remain under strict press embargo until 2 pm Eastern Time on the date of publication. For more information, please contact onepress@plos.org.

You will receive an invoice from PLOS for your publication fee after your manuscript has reached the completed accept phase. If you receive an email requesting payment before acceptance or for any other service, this may be a phishing scheme. Learn how to identify phishing emails and protect your accounts at https://explore.plos.org/phishing.

If we can help with anything else, please email us at customercare@plos.org.

Thank you for submitting your work to PLOS ONE and supporting open access.

Kind regards,

PLOS ONE Editorial Office Staff

on behalf of

Dr. Yi Cao

Academic Editor

PLOS One
